# Metagenomic‐based impact study of transgenic grapevine rootstock on its associated virome and soil bacteriome

**DOI:** 10.1111/pbi.12761

**Published:** 2017-08-09

**Authors:** Jean‐Michel Hily, Sandrine Demanèche, Nils Poulicard, Mélanie Tannières, Samia Djennane, Monique Beuve, Emmanuelle Vigne, Gérard Demangeat, Véronique Komar, Claude Gertz, Aurélie Marmonier, Caroline Hemmer, Sophie Vigneron, Armelle Marais, Thierry Candresse, Pascal Simonet, Olivier Lemaire

**Affiliations:** ^1^ INRA SVQV UMR‐A 1131 Université de Strasbourg Colmar France; ^2^ Laboratoire Ampère (CNRS UMR5005), Environmental Microbial Genomics École Centrale de Lyon Université de Lyon Ecully France; ^3^ IRD Cirad, Univ. Montpellier IPME Montpellier, France; ^4^ UMR 1332 Biologie du Fruit et Pathologie INRA Université de Bordeaux Villenave d'Ornon Cedex France; ^5^ Present address: European Biological Control Laboratory USDA‐ARS Campus International de Baillarguet CS 90013 Montferrier‐Sur‐Lez 34988 Saint Gely‐Du‐Fesc Cedex France

**Keywords:** environmental microbiology, metagenomics, grapevine, transgenic rootstock, safety and regulatory affairs

## Abstract

For some crops, the only possible approach to gain a specific trait requires genome modification. The development of virus‐resistant transgenic plants based on the pathogen‐derived resistance strategy has been a success story for over three decades. However, potential risks associated with the technology, such as horizontal gene transfer (HGT) of any part of the transgene to an existing gene pool, have been raised. Here, we report no evidence of any undesirable impacts of genetically modified (GM) grapevine rootstock on its biotic environment. Using state of the art metagenomics, we analysed two compartments in depth, the targeted *Grapevine fanleaf virus* (GFLV) populations and nontargeted root‐associated microbiota. Our results reveal no statistically significant differences in the genetic diversity of bacteria that can be linked to the GM trait. In addition, no novel virus or bacteria recombinants of biosafety concern can be associated with transgenic grapevine rootstocks cultivated in commercial vineyard soil under greenhouse conditions for over 6 years.

## Introduction

Until the development of genetic transformation tools, genetic improvement of plants relied solely on the selection of the most interesting genotypes, on crossing two individuals and thereby combining their genomes. The potential impact of the new genotype generated through classical breeding has largely been overlooked, as long as the final product obtained displayed the desired characteristics. From the beginning, and especially for plants, the impact of genetically modified (GM) organisms on all components of their environment has been considered as an important biosafety issue. Impacts have been extensively studied (Brookes and Barfoot, 2015; Devos *et al*., 2014; Scorza *et al*., 2013; Vàzquez‐Salat, 2013), and especially the risk of horizontal gene transfer (HGT).

While other transformation methods have been developed (Gordon and Ruddle, 1981; Jaenisch and Mintz, 1974; Klein *et al*., 1987; Neumann *et al*., 1982), the fundamentals of genetic engineering are based on a natural mechanism harnessed by *Agrobacterium* species*,* eventually resulting in a disease known as crown gall (Smith and Townsend, 1907) that affects a wide range of plants worldwide, including grapevine. *In fine*, part of the genetic make‐up of the bacteria, the transfer DNA (T‐DNA) (Chilton *et al*., 1977), is transferred and integrated in the genome of the plant. Recently it has been shown that natural GM crops have been grown and used for consumption for millennia, as all cultivated sweet potato accessions contain and express one or more T‐DNA sequences, while close wild relatives do not (Kyndt *et al*., 2015). This suggests that T‐DNA insertion(s) may have contributed to trait(s) selected during domestication. This case of HGT is far from being unique as endogenous viral elements have been described in many animal, fungus and plant species, including grapevine (Bertsch *et al*., 2009; Feschotte and Gilbert, 2012; Katzourakis and Gifford, 2010; Koonin, 2010). Plants appear to be both donors and recipients of horizontally mobilized genes (Bock, 2010), underlying the key role of HGT in evolution and genetic diversification (Soucy *et al*., 2015). Also, events of recombination between virus‐derived transgene transcripts and infecting viral RNAs have been described under laboratory conditions of high selection pressure (Borja *et al*., 1999; Gal *et al*., 1992; Greene and Allison, 1994; Morroni *et al*., 2009). Similarly, the transfer of transgenic sequences from plants to bacteria has been observed with specifically selected donor and recipient organisms (Kay *et al*., 2002). Yet, HGT events between GM plants and virus or bacteria populations should be observed in the field under conditions of natural selection pressure (Badosa *et al*., 2004; Capote *et al*., 2007; Demanèche *et al*., 2008; Vigne *et al*., 2004b).

The present investigation is aimed at investigating HGT in the biotic environment of GM perennial plants over a span of at least 6 consecutive years. The work was performed with GM grapevine rootstocks (Vigne *et al*., 2004b) expressing the coat protein gene of *Grapevine fanleaf virus* (GFLV) strain F13 (*F13‐cp*) and the neomycin phosphotransferase (*nptII*) gene, one of the most widely used selectable marker genes in plant genetic engineering (Jelenic, 2003; Turrini *et al*., 2015). These GM rootstocks were developed in an effort to confer resistance to GFLV (Lomonossoff, 1995; Sanford and Johnston, 1985). This virus is responsible for fanleaf degeneration, the most severe viral disease affecting vineyards worldwide (Andret ‐ Link *et al*., 2004). GFLV is transmitted by a soil‐borne ectoparasitic dagger nematode (*Xiphinema index*) and belongs to the genus *Nepovirus*. Its genome is composed of two single‐stranded positive‐sense RNAs that are sometimes associated with a satellite RNA (satRNA). In our experimental system, GM and control grapevines were planted in commercial vineyard soil infested with *X. index* and GFLV that was in a confined greenhouse. The virus was detected in all plants within the 4th year of plantation, implying that GFLV replicated within a transgenic background potentially interacting with transgenic *F13‐cp* derived transcripts and/or proteins. Our system is, therefore, an excellent model for addressing HGT in a perennial crop, by comparing the structure and genetic diversity of GFLV populations as well as rhizosphere bacterial populations using metagenomics approaches.

## Results

### No transgene‐derived sequences were detected in rhizospheric soil bacterial populations

**Figure 1:**
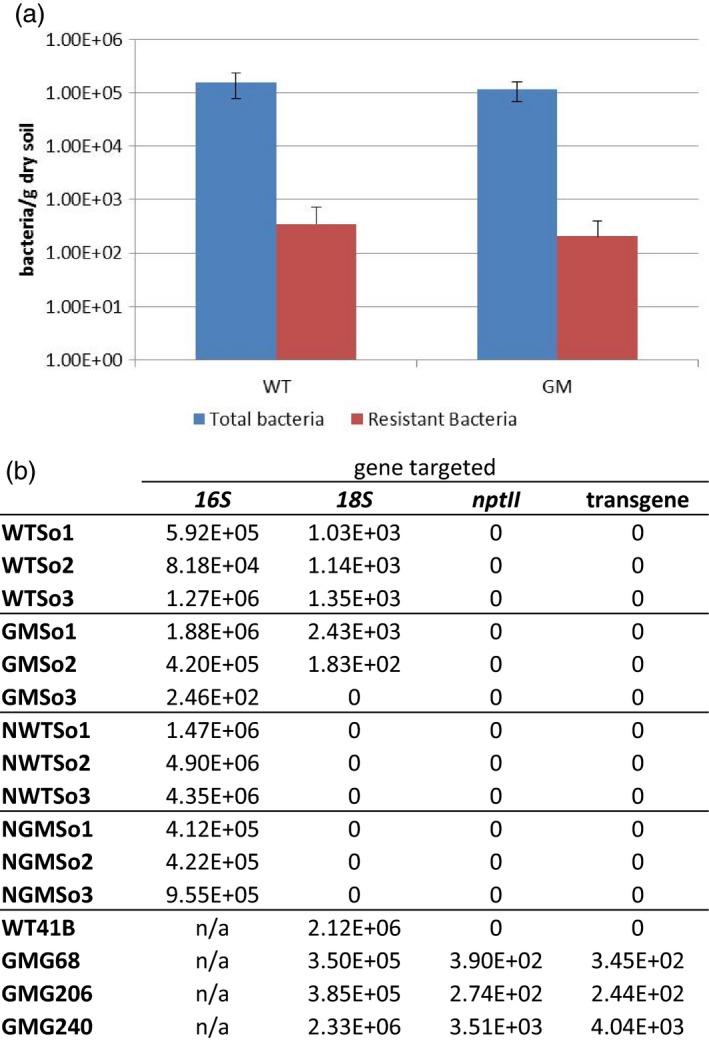
Antibiotic resistance of soil bacteria and gene transfer detection. (a) Number of total and Kanamycin‐resistant cultivable soil bacteria associated with Genetically Modified (GM) or Wild‐Type (WT) grapevine rootstocks. Results are expressed as the mean ± 95% confidence interval (2.31xSEM, for *n *= 9). (b) Gene quantification estimated by qPCR. Quantification was expressed as copies per ng of DNA for soil samples (WTSo1 to GMSo3) and for plant samples (WT41B to GMG240) and as copies per g of dry soil for Nycodenz^®^ samples (NWTSo1 to NGMSo3). Not applicable, n/a.

To investigate HGT between GM plants and bacterial populations in the rhizospheric soil, antibiotic resistance in soil bacteria sampled from GM expressing *F13‐cp* and *nptII* (Vigne *et al*., 2004b) and wild‐type (WT) grapevine rootstocks was characterized using two different approaches: (i) a classical isolation and culture‐based approach and (ii) direct molecular‐based methods, such as PCR, qPCR and high‐throughput sequencing. The culture‐based approach relies on a direct counting of bacteria growing on culture media supplemented with or without kanamycin to estimate the cultivable antibiotic‐resistant or total bacteria, respectively. The commercial vineyard soil from Bergheim used in this study contained an average of 10^5^ cultivable bacteria per gram of dry soil (Figure 1a) of which 0.18% displayed resistance to kanamycin. This percentage represented approximately 300 cultivable kanamycin‐resistant bacteria per gram of dry soil. No significant difference between rhizospheric soil of GM and WT grapevines was observed in either total or kanamycin‐resistant cultivable bacteria (Mann–Whitney test, *P *=* *0.440 and 0.930, respectively). Kanamycin‐resistant bacteria were further tested by PCR for the presence of transgene sequences with two sets of primers targeting *nptII* (nptII666/668) and a specific sequence of the transgene construct (TgLarge), respectively (Figure S1). Of the 1,128 individual kanamycin‐resistant bacterial cultures tested, no amplification was obtained with either primer set, whereas the *16S rRNA* gene (used as a control) was successfully amplified from all bacteria samples (Figures 1b and S1b). Furthermore, qPCR and DNAseq were applied to total and bacterial DNA from soil samples, bypassing potential limitations associated with the ability to culture bacteria. Total soil DNA samples contained 1.8 × 10^2^ to 2.4 × 10^3^ copies/ng DNA of *18S rRNA*, as shown by qPCR, whereas no *18S rRNA* (used as a marker for eukaryotic DNA presence) was amplified in any of the total bacteria DNA samples tested, as expected (Figure 1b). From both total and bacterial DNA, no transgene (Tgshort) or *nptII* (*nptII*sens) sequences (Figure S1a) were amplified. In contrast, plant DNA samples from transgenic grapevines contained 2.4 × 10^2^ to 4.0 × 10^3^ copies/ng DNA of *nptII* and transgene sequence, while 3.5 × 10^5^ to 2.3 × 10^6^ copies/ng DNA of *18S rRNA* were identified (Figure 1b). This corresponded to a ratio of 1 copy of transgene for about 100 to 1000 copies of 18S. At the same time, total DNA from six soil samples, three exposed to GM grapevines (GMSo) and three exposed to WT grapevines (WTSo), were submitted to Miseq high‐throughput sequencing and direct mapping of reads against the genome of *Agrobacterium tumefaciens* strain LBA4404 (WGS accession number JMKN01000001‐39) and pBin19 (GenBank number U12540.1). Both, the binary plasmid and the *A. tumefaciens* strain, had been used to generate GM rootstocks. The 8.7 million DNA sequences data set was analysed by applying high stringency (0.99 read length and 0.99 sequence similarity) to avoid false positive hits from soil bacteria DNA. Not a single soil DNA read corresponding to *A. tumefaciens* strain LBA4404 or pBin19 was detected. This was still the case even when the stringency was reduced to 0.5/0.8 (read length/similarity). Identical results were obtained with RNAseq of leaf samples from GM grapevines, validating the fact that plant (T0 transformants) and soil samples did not contain, at detectable levels, the recombinant bacterial strain used for transformation.

### No transgene‐derived sequences translocation to scion or to viral population was observed

GM and WT rootstocks were exposed to a natural GFLV infection. To examine HGT between GM grapevines and GFLV populations, high‐throughput sequencing was used, and two data sets were analysed as follows: (i) RNAseq data from 26 samples [corresponding to four sample categories collected from GM grapevine rootstock line G68 (GMR), wild‐type grapevine rootstock (WTR), wild‐type scion grafted onto GMR (ScGM) and wild‐type scion grafted onto WTR (ScWT)] and (ii) high‐throughput data from immunocapture‐RT‐PCR (IC‐RT‐PCR) products corresponding to encapsidated GFLV RNAs. To detect reads corresponding to transgene transcripts derived from *F13‐cp* or *nptII*, high stringent mapping parameters were used to avoid potential cross‐mapping with reads derived from the infecting‐GFLV population and/or endophytic bacterial communities. No reads obtained by RNAseq matched the transgenic *F13‐cp* sequence in any of the samples other than those from the transgenic rootstock (Tables 1 and S1, stringency 0.97/0.99). Similar results were obtained when investigating the *nptII* sequence, with reads being detected only in GMR samples (Tables 1 and S1, stringency 0.97/0.99). These results, therefore, reveal no detectable transfer of transgene‐derived transcripts through the graft union as no transgene sequence signature was recovered from any of the ScGM samples (leaf or inflorescence, Table S1). These observations also suggest that no replication or movement‐competent GFLV recombinant containing transgene‐derived sequences emerged in the transgenic grapevine rootstocks with subsequent translocation into the scion. These particular findings were confirmed by IC‐RT‐PCR‐NGS with no detection of transgenic *F13‐cp* reads in any of the samples tested (GM or non‐GM, Tables 1 and S1). Such a conclusion was not drawn due to a limitation of the approaches to detect recombination events since many crossover sites were identified in both GFLV RNA1 and RNA2 [including the coat protein (*cp*) sequence] of the natural infecting‐GFLV populations (see section below on natural GFLV recombinants identification). After removal of transgene‐derived reads from the data set and relaxing mapping parameters (0.5/0.7), a large number of reads corresponding to GFLV *cp* were detected in all samples (Tables 1 and S1). This result reflected GFLV infection, unveiling the variability of the viral population (see next paragraph). While mapping at low stringency dramatically increased the number of GFLV reads recovered, such an increase in reads was not observed for *nptII* (Tables 1 and S1). This observation suggests that, similar to soil bacterial populations as mentioned above, endophytic bacteria in grapevine samples (see below and Figure S2) do not carry *nptII* sequences, significantly reducing any potential HGT events involving the GM‐derived *nptII* gene.

**Table 1:** Transgenic‐derived transcripts detection and sanitary status using RNAseq. Numbers are average of reads directly mapped onto transgenic *F13‐cp* and *nptII* sequences, from all WTR (wild‐type rootstock), ScWT (scion grafted onto WTR), GMR (transgenic rootstock) and ScGM (scion grafted onto GMR) samples from leaf tissue. Virus and viroid presence are expressed in RPKM: Reads Per Kilobase per Million reads mapped to the reference

		Sample name	WTR	ScWT	GMR	ScGM
		Total trimmed reads	51 379 136 ± 4 580 482^b^	39 203 579 ± 7 454 413^ab^	30 368 183 ± 5 195 118^a^	36 263 138 ± 6 431 238^ab^
References Transgene	size (nt)	Stringency*				
*F13‐CP*	1 515	0.97/0.99	0.17 ± 0.17^a^	0.67 ± 0.42^a^	204.6 ± 48.99^b^	0.00 ± 0.00^a^
		0.5/0.7†	416 922 ± 42 119^b^	197 349 ± 44 058^a^	166 419 ± 34 989^a^	126 110 ± 15 537^a^
*nptII*	796	0.97/0.99	0.00 ± 0.00^a^	0.50 ± 0.50^a^	648.40 ± 139.76^b^	0.00 ± 0.00^a^
		0.5/0.7†	0.00 ± 0.00^a^	0.17 ± 0.17^a^	355.80 ± 72.27^b^	0.00 ± 0.00^a^
Virus/viroid						
GFLV RNA1	6 855	0.5/0.7†	2 142.46 ± 210.04^c^	1 095.74 ± 137.37^ab^	1 349.96 ± 197.86^b^	818.78 ± 83.30^a^
GFLV RNA2	3 333	0.5/0.7†	4 687.45 ± 654.53^b^	2 765.81 ± 360.86^a^	2 956.36 ± 424.20^a^	1 995.55 ± 230.72^a^
GFLV satRNA	1 004	0.5/0.7†	1 682.54 ± 364.25^b^	981.05 ± 166.56^a^	1 030.70 ± 150.24^ab^	628.84 ± 186.02^a^
GRSPaV	8 725	0.5/0.7†	82.50 ± 21.24^ab^	318.10 ± 80.81^b^	0.12 ± 0.04^a^	149.97 ± 83.98^ab^
HSVd	298	0.5/0.7†	75.00 ± 9.13^b^	142.06 ± 20.22^c^	0.24 ± 0.13^a^	176.35 ± 10.29^c^
GYSVd	366	0.5/0.7†	7.98 ± 7.98^b^	59.01 ± 11.61^c^	0.06 ± 0.06^a^	47.90 ± 6.37^c^

^a, b, c^Symbolize the post hoc analysis (Fisher's least significant difference, LSD).

*Denotes the stringency (minimum length of the read/ homology with the sequence) for mapping reads to sequence of interest.

†Indicates that transgenic *cp* and *nptII* reads were removed prior to performing analysis at a lower stringency.

### No specific soil bacterial communities' selection linked to GM grapevine

**Figure 2:**
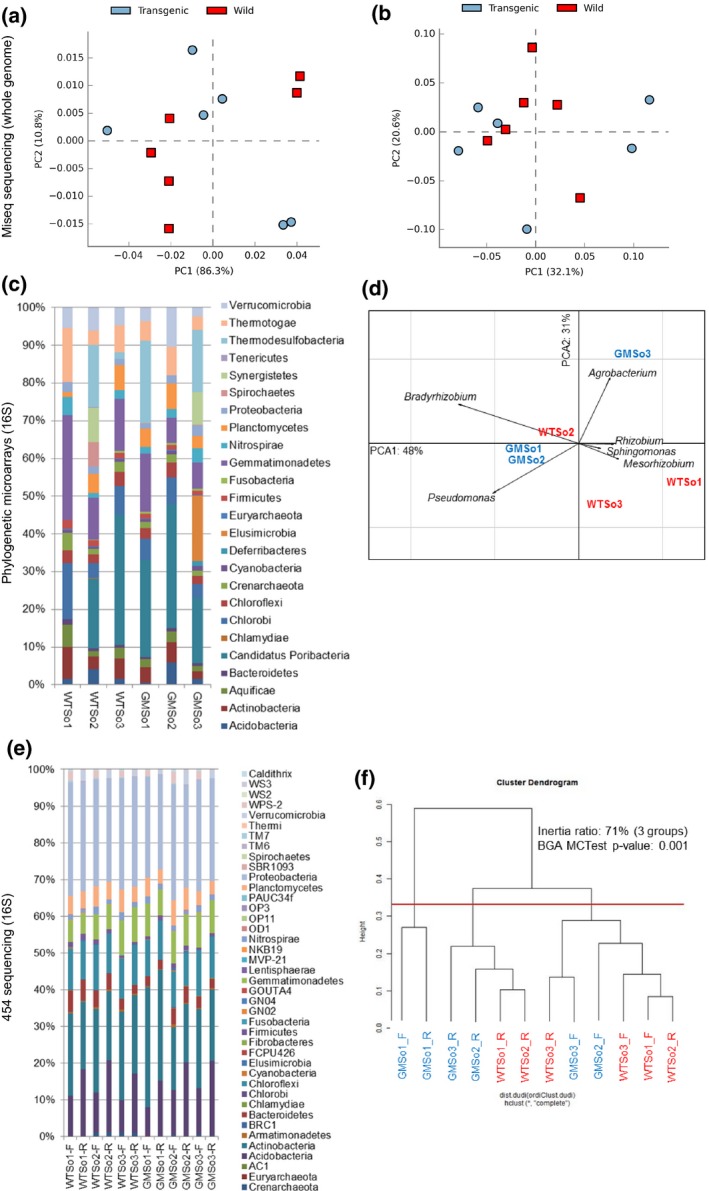
Microbial diversity comparison using Miseq whole‐genome sequencing (a and b), phylogenetic microarrays (c and d) and 454 16S rRNA gene sequencing (e and f). (a) Principal component analysis (PCA) on MG‐RAST organism abundance with representative hits classification at 80% cut‐off. (b) PCA on MG‐RAST functional abundance with hierarchical classification at 80% cut‐off. (c) Sample comparison among the 25 phyla represented on the microarray. (d) Sample comparison at the genus level by PCA. (e) Sample comparison at the phylum level (%). (f) Groups generated by clustering. WTSo1 to WTSo3: rhizospheric soil samples of wild‐type plants; GMSo1 to GMSo3: rhizospheric soil samples of genetically modified plants; _F : forward reads related to the V4 region of the 16S RNA gene; _R : reverse reads related to the V6 region of the *16S RNA* gene.

To characterize the diversity of bacterial communities in soil samples exposed to transgenic (GMSo) and wild‐type (WTSo) rootstocks, three metagenomics strategies were used. The first one was a DNAseq approach from which 8.7 million DNA sequences were analysed using MG‐RAST (http://metagenomics.anl.gov/, last visited 05/2017). No significant differences were observed between GMSo and WTSo soil samples regarding the type of organism and their abundance (ANOVA, *P *>* *0.05, Figure 2a,b). In addition, a microarray for *16S rRNA* detection based on 25 phyla showed some variability between samples (Figure 2c). For example, the *Candidatus*/*Poribacteria* phylum was not detected in sample WTSo1, whereas it represented 17.3% and 34.6% in samples GMSo3 and WTSo3, respectively. *Spirochaetes* were present at 6.4% in sample WTSo2, but only at 0.2% and 0.1% in samples GMSo1 and GMSo3, respectively, and they were absent in samples WTSo1, WTSo3 and GMSo2. Also, only samples WTSo2 and GMSo3 displayed *Synergistetes* (9.2% and 8.7%, respectively). All these differences were not significant when referring to GM and WT traits (ANOVA, *P *=* *0.840; *t*‐test WT *versus* GM, *P *=* *0.790). The principal component analysis at the genus level demonstrated that samples WTSo2, GMSo1 and GMSo2 grouped according to the first axis, representing 48% of the observed variability (Figure 2d). Again, no grouping was observed when considering the GM/WT trait. To further describe bacterial communities within soil samples, a 454 sequencing‐based approach of *16S rRNA* was performed (Beckman Coulter Genomics, V4‐V6 region). The dominant bacterial groups across all samples were *Proteobacteria*,* Actinobacteria*,* Acidobacteria*,* Chloroflexi*,* Gemmatimonadetes, Planctomycetes* and *Bacteroidetes* (Figure 2e). When considering all samples, a clustering was observed but not according to GM/WT trait (Figure 2f, BGA MC‐Test, *P *=* *0.001), as most GM samples grouped with WT samples. Interestingly, the sequencing region/primers effect was more discriminating than the GM *versus* WT grapevine traits (Figure 2f), and sample GMSo1 presented a higher proportion of *Actinobacteria* (Figure 2e,f). Bacterial signatures were also detected from leaf samples (Figure S1). At the genus level, no significant differences were detected when comparing GM/WT grapevines, (*P *> 0.050, Welch's two‐sided t‐test, with Benjamini‐Hochberg FDR multiple test correction, Figure S2a). When comparing bacterial composition in soil and leaf samples (GMR, ScGM, GMSo, WTR, ScWT and WTSo), 410 significant different components were detected (ANOVA with Tukey‐Kramer post hoc test and Benjamini‐Hochberg FDR multiple test correction). Among these components, endophytic bacteria affiliated to the genus *Staphylococcus* were significantly more abundant in leaf than in soil samples, whereas the *Streptomyces* and *Clavibacter* genera were more abundant in soil than in leaf samples (Figure S2b). While viral signatures were present in all leaf samples, none were detected in soil samples. As expected, eukaryotic sequences were much more abundant in leaf than in soil samples (Figure S2c).

### No evidence of undesired impact of transgenic expression on virome

**Figure 3:**
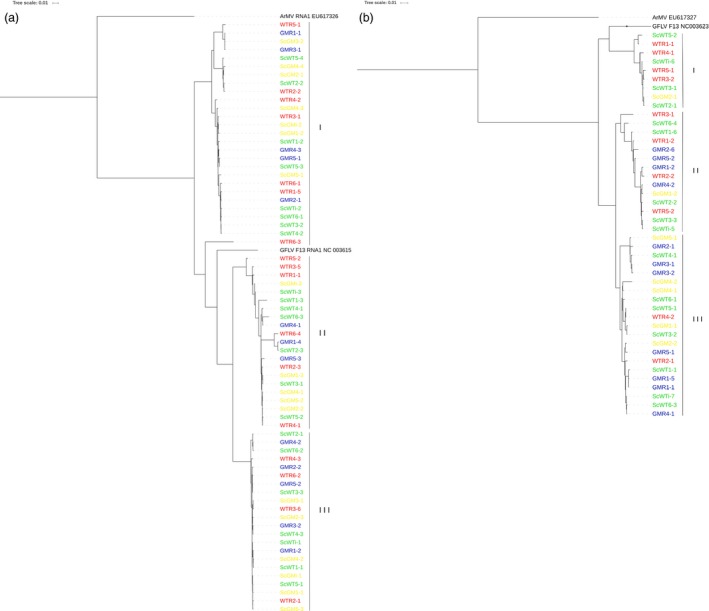
Phylogenetic relationships of GFLV RNA1 and RNA2 genomes obtained from leaf samples from GM rootstock (GMR, in blue), non‐GM rootstock (WTR, in red), scion grafted onto GMR (ScGM, in yellow) and scion grafted onto WTR (ScWT, in green) samples assembled with CLC Workbench 8.5.1 software. Phylogenetic tree based on the Maximum likelihood of (a) 70 full‐length sequences of GFLV RNA1 and (b) 44 full‐length sequences of GFLV RNA2. Bootstrap values are shown.

**Figure 4:**
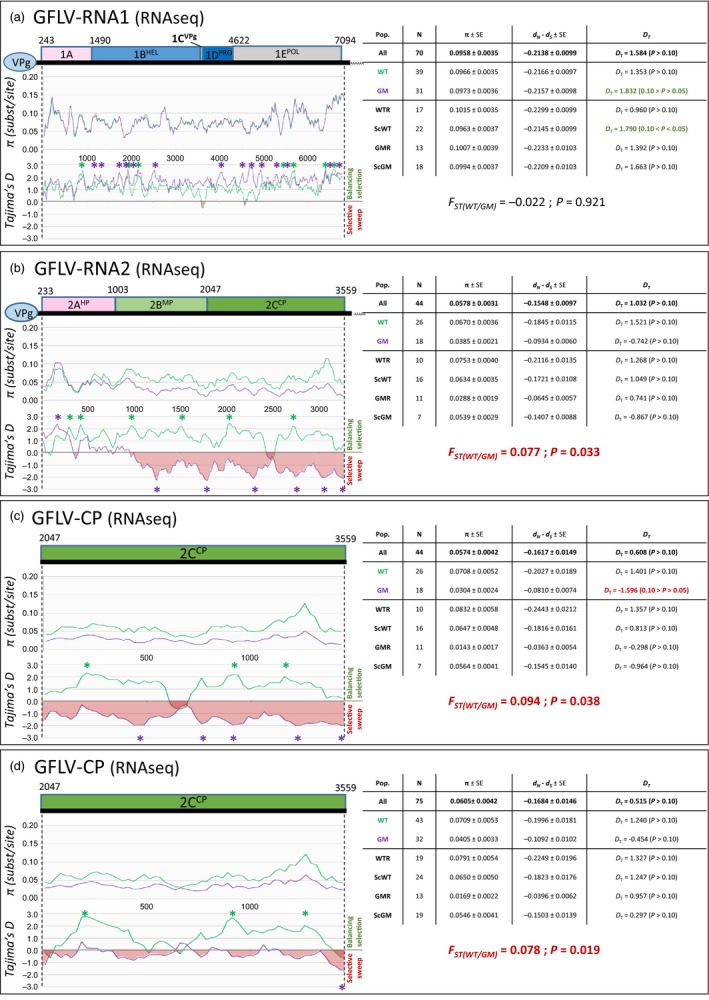
Genetic diversity analyses of GFLV RNA1 sequences (a), GFLV RNA2 sequences (b), GFLV *cp* gene sequences (c) and all GFLV sequences spanning the *cp* gene (d) assembled with CLC Workbench 8.5.1 software. Sequences were obtained from GM rootstock (GMR), non‐GM rootstock (WTR), scion grafted onto GMR (ScGM) and scion grafted onto WTR (ScWT) samples. Graphics represent π (substitution per site) along each sequence and Tajima's D (*D*_*T*_) for evolution study, comparing GM grapevine‐related sequences (GMR + ScGM, in aquamarine) and WT grapevine‐related samples (WTR + ScWT, in purple). Overall genetic diversity is composed of N: number of sequences, π ± SE: overall genetic diversity (substitution/site) ± standard error, *d*_*S*_,*d*_*N*_: diversity of synonymous and nonsynonymous substitutions, respectively (*d*_*N*_ *‐* *d*_*S*_ < 0: negative/purifying selection; *d*_*N*_ *‐* *d*_*S*_  =  0: neutral selection; *d*_*N*_ *‐* *d*_*S*_ > 0: positive/diversifying selection), Tajima's D (*D*_*T*_): *D*_*T*_ = 0 correspond to a mutation‐drift equilibrium, *D*_*T*_ > 0 indicates balancing selection, sudden population contraction and *D*_*T*_ < 0 distinguish a recent selective sweep, population expansion after a recent bottleneck, (* P < 0.05). Genetic differentiation expressed as the Fixation Index (*F*_ST_) either overall or pairwise, yielding the genotypic frequencies in the entire population, with associated p‐value. Significant p‐values are in red.

A comprehensive virological evaluation was performed, and the molecular diversity of the three GFLV RNAs (genomic RNAs 1, 2 and satellite) was assessed for each plant tested. For this, a dual strategy was used as follows: (i) a direct mapping of reads against a collection of reference sequences of grapevine‐infecting viruses and viroids species and (ii) a *de novo* assembly of reads followed by BlastN/BlastX annotation of contigs. Remarkably, other than for GFLV‐related reads, GM samples did not display any other viral reads (only extremely low contamination level with RPKMs = 0, Tables 1 and S2). On the other hand, in all nontransgenic samples (e.g., WTR, ScWT and ScGM), reads matching *Grapevine rupestris stem pitting‐associated virus* (GRSPaV), *Hop stunt viroid* (HSVd) and *Grapevine yellow speckle viroid* (GYSVd) were readily detected in addition to GFLV (RNA1, RNA2 and satRNA) reads (Tables 1 and S2). The absence in GM samples of GRSPaV, HSVd and GYSVd, which are extremely widespread grapevine pathogens, likely results from their elimination through somatic embryogenesis, which is the cornerstone of the grapevine transformation process (Gambino *et al*., 2006; Panattoni *et al*., 2013). While GFLV genomic RNAs and satRNA were detected in all samples, rootstock samples presented greater RPKM values (e.g., virus titre) than scions grafted onto them, regardless of the viral RNA (ANOVA test, *P *≤* *0.0421, Table S2). Even though infected with GFLV, GM rootstocks accumulated less virus than WTR samples (ANOVA test, *P *=* *0.024, 0.064 and 0.159, for RNAs 1, 2 and satellite, respectively, Table 1). From the direct mapping analyses, a single‐nucleotide polymorphism (SNP) study of GFLV genomic RNAs and satellite RNA was performed. Various SNP detection levels were tested in order to check different depths of the variability spectrum: from very deep (with detection limit being set at 0.5%), up to the detection of SNPs being the most represented (80%), with two levels in between (20% and 51%). Quantitatively, while SNP numbers fluctuated among samples (Table S3), no statistical differences (ANOVA test, *P *≥ 0.0744) were found between sampling categories in each detection level tested, within any of the GFLV RNAs or the *cp*. To further investigate GFLV population diversity, a *de novo* assembly was performed. From the RNAseq data set, seventy complete GFLV RNA1 sequences (GenBank accessions KX011072‐KX011075 and KX034840‐KX034905) were obtained. A phylogenetic analysis of the RNA1 sequences grouped them into three distinct clades (Figure 3a) in which all sampling categories [GM‐related samples (GMR and ScGM) or WT‐related samples (WTR and ScWT)] were represented. This suggests no selection for a particular virus population among the different sampling categories (Chi‐squared test, *P *=* *0.976). In addition, the genetic diversity (π = 0.0966 ± 0.0035 and 0.0973 ± 0.0036 for WT‐ and GM‐related populations, respectively), the ratio of synonymous and nonsynonymous substitution (*d*_N_ *–d*_S_ = −0.2166 ± 0.0097 and −0.2157 ± 0.0098, respectively) and the Tajima's D values (*D*_T_ = 1.353 and 1.832, respectively) followed the same pattern between WT and GM populations, suggesting no particular effect of the GM grapevine rootstocks on the GFLV RNA1 diversification and selection. This was supported by the very low value of the fixation index *F*_ST_ between the WT and GM populations (*F*_ST_ = −0.022; *P *=* *0.921), supported no genetic differentiation between these two populations (Figure 4a). Similarly, 31 full‐length sequences of GFLV satRNA were obtained (GenBank accessions KX034950‐KX034980). While all sequences clustered together (clade I, Figure S3a) compared to the known satellite diversity (Čepin *et al*., 2016; Gottula *et al*., 2013), no effect of the GM plants on the satRNA diversity and selection was observed, as confirmed by the absence of genetic structure between GM and WT populations (Figure S4a, *F*_ST_ = −0.013; *P *=* *0.645). As for GFLV RNA2, 44 complete sequences (GenBank accessions KX034906‐KX034949) were assembled, alongside a total of 75 partial sequences spanning the 1515 bp of the *cp*. Phylogenetic analyses of the RNA2 and *cp* sequences grouped them into three clades (Figures 3b and S3b, respectively). Interestingly, the three clades did not display members from each sampling category as GMR sample sequences were not accounted for in clade I (Figures 3b and S3b). This observation indicated a potential population imbalance, but the existence of a selective bias linked to the presence of the *F13‐cp* transgene from the GMR category was not statistically supported (Chi‐squared test, *P *=* *0.208 and *P *=* *0.135 for the RNA2 and *cp* data sets, respectively). Nonetheless, the genetic diversity was drastically lower in GM than WT populations (Figure 4b; 0.0385 ± 0.0021 and π = 0.0670 ± 0.0036, respectively). In addition, Tajima's D test was significantly negative for the GM population (Figure 4b; *D*_T_ = −0.742), underlying a recent selective sweep of the *cp* in particular, while WT population followed a balancing selection (*D*_T_ = 1.521). Furthermore, the *F*_ST_ values indicated a significant genetic differentiation between GM and WT populations for both RNA2 and *cp* (Figure 4b,c,d; *F*_ST_ = 0.077; *P *=* *0.033 for RNA2 data set; *F*_ST_ = 0.094, *P *=* *0.038 and *F*_ST_ = 0.078, *P *=* *0.019 for *cp* data sets). More precisely, when testing sampling classes in a pairwise comparison, a number of *F*_ST_ with statistical significance were observed, in each case involving the GMR category (Table S4). These results clearly demonstrate that the RNA2 and especially the *cp* gene are distinct between WT‐ and GM‐related populations (Figure 4b,d). Remarkably, this difference in *cp* structuration was only due to the clade I missing from GMR class, as removal of clade I sequences from the data set abolished the genetic differentiation between populations (Figure S4b; *F*_ST_ = −0.020; *P *=* *0.474). These results were confirmed by performing the same analyses on the IC‐RT‐PCR data set (Figures S3c and S4c and Table S4).

### Detection of viral recombination events, none involving transgenic sequence

**Figure 5:**
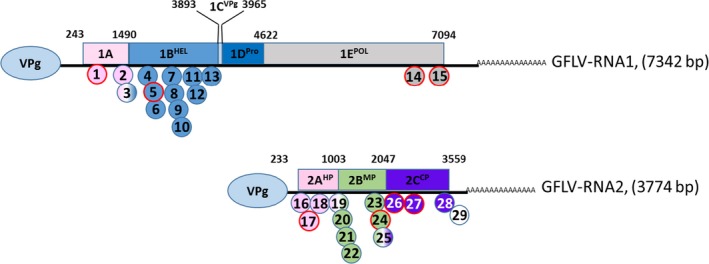
Localization of recombination sites detected in GFLV RNA1 and RNA2 sequences obtained from *‘de novo*’ assembly using CLC Genomics Workbench software (positions based on GFLV‐F13 RNA1 and RNA2 sequences, NC003615 and NC003623, respectively). Colour code indicates gene sequences that have undergone recombination in each viral genome. In red are shown the sites that were analysed and confirmed to be biologically present in the viral population. Numbers correspond to those in Table S6.

In previous work (Vigne *et al*., 2004b), despite the fact that many natural recombination events were detected (Vigne *et al*., 2004a, 2005), no HGT between *F13‐cp* transgene‐derived transcripts and GFLV populations was identified. In this study, of the 70 complete RNA1 sequences (mentioned above), 15 recombination sites were identified (Figure 5 and Table S5, with corrected *P*‐value ≤5.59 × 10^e−12^). While no recombination events were detected in the *Vpg* or *protease* sequences, between two and 11 breakpoints were identified in the *1A*,* helicase* and *polymerase* sequences. All sites were confirmed in the RNASeq data set when recombinants were used as references for a direct mapping analysis using very stringent parameters (Table S5). Of these, four randomly selected crossover sites were confirmed as being biologically genuine in the viral population following RT‐PCR and Sanger sequencing. The same investigation was performed using the complete set of full‐length RNA2 sequences, and 14 recombination sites (corrected *P*‐value ≤4.10 × 10^e−09^) were identified encompassing all three RNA2 coding regions (Figure 5 and Table S5). Using the larger *cp* data set, four recombination events were identified, two of which, randomly selected, were confirmed to be biologically present in the viral populations. As previously documented (Vigne *et al*., 2004b), none of the crossover events identified within the *cp* were associated with the *F13‐cp* transgene sequence.

## Discussion

It is now widely accepted that horizontal gene transfer (HGT) is shaping the web of life (Soucy *et al*., 2015). The selectable marker *nptII* that confers resistance to kanamycin is present in a large number of GM crops and has been extensively studied (Dantas *et al*., 2008; Jelenic, 2003). Two decades ago it was concluded that *nptII* poses no risk to humans, animals or the environment (Fuchs *et al*., 1993; Nap *et al*., 1992). The results presented here demonstrate that the presence of *nptII* in GM grapevine rootstocks did not increase the level of kanamycin‐resistant bacteria in the rhizospheric soil because similar amounts of kanamycin‐resistant bacteria were detected in soil samples of WT rootstocks. This confirms previous results on the occurrence of bacteria resistant to kanamycin in various ecosystems (D'Costa *et al*., 2007; Dantas *et al*., 2008; Li *et al*., 2009). Also, *nptII* was not detected in DNA from soil samples by qPCR or NGS even after a six‐year period of continuous GM grapevine cultivation. In addition to the transgene flanked by the left and right T‐DNA borders, fragments from the tumour‐inducing plasmid and larger fragments, up to 18 kb, of *Agrobacterium* chromosomal DNA can occasionally be integrated into the plant genome during *Agrobacterium tumefaciens*‐mediated transformation (Ulker *et al*., 2008), potentially increasing the risk of HGT. Within our different NGS data sets, sequences of the *A. tumefaciens* strain used for grapevine transformation were never detected in DNA recovered from rhizospheric soil sampled from around GM rootstocks, in leaf samples or within the genome of GM grapevine rootstocks. Additionally, while differences in microbial populations were observed among soil samples subjected to various conditions, those were due to biological sample variations, and not to the presence of GM rootstocks. More importantly, similar dominant bacterial groups have been observed in American vineyard soils (Burns *et al*., 2015; Zarraonaindia *et al*., 2015) as well as in our study, although differences in abundance were noticed. For example, *Proteobacteria*,* Actinobacteria* and *Acidobacteria* were the three most abundant bacteria in our traditional vineyard soil and in Californian soils (Burns *et al*., 2015), whereas *Bacteroidetes* were in the top three in New York soil (Zarraonaindia *et al*., 2015) but were less abundant in our study (7th most abundant). No specific bacterial groups seemed to be selected for in either WT or GM rhizospheric soils. These data support the role of soil properties and crop management practices in shaping the composition of microbial and fungal communities and their diversity (Campisano *et al*., 2014; Pancher *et al*., 2012), with no detectable effect of GM rootstocks.

As expected, transgene‐derived transcripts were detected only in GM samples and were not found in any WT scions grafted onto GM rootstocks. Furthermore, as for bacteria, no HGT events between the *F13‐cp* transgene sequence and GFLV populations were identified, while a large number of natural recombination events were readily detected *in silico* and *in vivo*, confirming previous studies (Vigne *et al*., 2004a, 2005). When added to previous investigations (Tepfer *et al*., 2015), these findings, using state of the art high‐throughput sequencing technology, confirm that GM grapevine did not result in the emergence of novel recombinant viral isolates. Nonetheless, the GM grapevine line investigated in this study impacted the GFLV population diversity. Such a specific effect on the viral RNA2/*cp* population, observed only in the GMR category, could potentially be explained by a transgene‐mediated silencing mechanism specifically targeting homologous *cp* sequences. Two additional elements strengthen this hypothesis: (i) GMR, here G68 line, is a GM rootstock presenting multiple transgene inserts (Vigne *et al*., 2004b), potentially leading to the production of *F13‐cp* dsRNA, a major trigger of silencing (Meister and Tuschl, 2004; Wesley *et al*., 2001), and (ii) the counter‐selected RNA2 population from clade I (Figure 3b) is the closest to the *F13‐cp* transgene sequence (Figures 3b and S3b), suggesting a homology‐dependent exclusion by gene silencing mechanism. This hypothesis is re‐enforced by the fact that while the level of substitutions per site (π) is lower in GM than WT population, the ratio of nonsynonymous/synonymous substitutions (*d*_*N*_‐*d*_*S*_) is similar in both populations, suggesting that the selection does not occur at the protein level. These results were consistent not only in the GM rootstock but also in most (4/5) of the scions grafted onto it, increasing the potential usefulness of the rootstock‐mediated resistance technology against a telluric viral agent such as *Grapevine fanleaf virus*.

Environmental safety concerns have been raised on the release of GM plants. These risks could be particularly pertinent in the case of a perennial crop, such as grapevine, as vineyards are worked for decades. Our study strongly suggests that GM grapevine rootstock cultivation does not favour the development of recombinant viruses and/or endophytes of biosafety concern nor disturb the composition of nontargeted rhizospheric bacterial communities. Also, transgenic‐derived transcripts recovery was limited to GM tissues and was not detected in WT scion grafted onto GM rootstocks. Taken together, all these results could potentially guide policy makers when deciding about GM rootstock regulation, likely shaping the agriculture of tomorrow. Such comprehensive multiscale environmental impact study of a GM crop should be performed not only in a mesocosm environment (represented by a confined greenhouse with high‐density planting) but also in open‐field trials where HGT events have yet to be observed and where abiotic conditions might distinctly modify the selection and evolution of virus and/or bacteria communities.

## Experimental procedure

### Plant material, soil samples and conditions

In this work, five genetically modified (GM) grapevine rootstock lines (G68, G77, G206, G219 and G240) (Vigne *et al*., 2004b) were used. For the bacterial genomic analysis, soil surrounding all GM line rhizospheres was tested; however, in the viral genomic study, only the G68 GM line was fully investigated. While presenting different insertion numbers and sites (Vigne *et al*., 2004b), all GM lines were transformed with the *coat protein* (*cp*) gene of *Grapevine fanleaf virus* (GFLV) strain F13 (*F13‐cp*) in sense under the 35S promoter and the *npt*II gene under nos promoter, which was used as the selective marker gene (Figure S1a). To test potential movement of transgenic molecules intraplant through conduit‐vessels, ‘Pinot meunier’ grape variety was grafted onto GM and non‐GM rootstocks (41B rootstocks, same variety as the GM lines). Five repeats of each GM line and six repeats of untransformed controls, grafted or not, were randomly planted (Figure S5) in September 2006 in a homogenized commercial vineyard soil (Bergheim, France, 48.199405 lat., 7.349196 long.), infested with nematode *Xiphinema index* and GFLV. The soil was transported to the INRA experimental station in Colmar (The Local Monitoring Commitee *et al*., 2010), and was kept in a greenhouse (48.064457 lat., 7.334899 long.) within a 6 × 2 × 0.5 m confined arena made of concrete. Emergence and spread of the disease were monitored every year. In this setting, we studied the impact of GM plants on telluric microbiota and on the natural GFLV population *in planta*, using metagenomics sequencing analyses.

For the study on the metavirome, two different leaf samplings were performed; all completed in spring/early summer, when the virus titre is believed to be at its peak (Čepin *et al*., 2010; Walter and Etienne, 1987; Walter *et al*., 1984). In 2013, leaf samples were collected from G68‐GM Rootstock (GMR), non‐GM Rootstock (WTR), Scion grafted onto GMR (ScGM) and Scion grafted onto WTR (ScWT) from which IC‐RT‐PCR‐based NGS analyses were performed (Figure S5). In 2015, RNAseq‐based analyses were performed on the same plants.

For the soil microbiota genomic study, three samples of 1.5 kg of soil were collected in October 2012 along WT rootstocks rooting system (WTSo1, WTSo2 and WTSo3) as well as along roots of three GM rootstocks (GMSo1, GMSo2 and GMSo3) (Figure S5). Samples were immediately sieved through a 2 mm mesh, providing for about 700 g soil for each sample, that were sent overnight to École Centrale de Lyon, Ecully (France) and stored at 22 °C in sterile plastic 2‐L flasks. For a detailed experimental procedure, see Appendix S1.

### Bacterial genomics and soil study

#### Isolation of antibiotic‐resistant bacteria

Total cultivable and antibiotic‐resistant bacteria were isolated from the six soil samples described above. Soil suspensions were serially diluted for total cultivable bacteria counts. For antibiotic‐resistant bacteria counts, soil suspensions were spread on medium supplemented with 50 μg/mL kanamycin and 50 μg/mL neomycin. Three Petri dishes per dilution were used, and soil dispersion was spread in triplicate for each of the six soil samples.

#### Bacterial cells recovery

Telluric bacteria were extracted from the six soil samples (see above, and named NWTSo1, NWTSo2, NWTSo3, NGMSo1, NGMSo2 and NGMSo3) using a Nycodenz^®^ gradient (Axis‐shield, Oslo, Norway).

#### Soil DNA extraction

Total DNA was extracted from the six soil samples (Total RNA Isolation Kit with DNA elution accessory kit, Mo Bio Laboratories, Carlsbad, CA) as per manufacturer's instructions. Concentrations were determined with Qubit^®^ 2.0 fluorometer (London, UK) and were stored at −20 °C.

#### PCR amplifications

The different targeted genes, primers set, annealing temperature, time of elongation and PCR product sizes are shown in Figure S1b. PCR amplifications were performed using the Titanium Taq Polymerase (Clontech Laboratories Inc., Palo Alto, CA). PCR amplifications to prepare standards for qPCR were performed using the Invitrogen Taq Polymerase (Invitrogen, Carlsbad, CA) with appropriate primers (Edwards *et al*., 1989; Fierer *et al*., 2005) (Figure S1b).

#### Quantitative PCR assays

Quantitative PCR was performed using the SYBR Green chemistry. The different targeted genes and other information are described in Figures 1 and S1. PCR amplifications were performed with SensiMix SYBR No‐ROX Kit (Bioline, Reagents Ltd, London, UK). Amplifications were carried out using the Rotor‐gene 6000™ (QIAGEN, Courtaboeuf, France). The real‐time PCR (qPCR) reaction mixture yielded a final volume of 20 μL including 1X SensiMix ™ SYBR qPCR Kit, 0.8 μL of each primer at a concentration of 10 μm, 2 μL of DNA (samples and standards), Nycodenz^®^ samples or water (*q.s.p*. 20 μL). Gene copy numbers of samples were determined according to standard curves with *R* ^2 ^> 0.99, obtained from serial dilutions of purified PCR products (10‐fold dilutions from 10^9^ to 10^2^ copies were used). Data were analysed with Rotor‐Gene 6000 software. The threshold limits were manually positioned at the beginning of the exponential phase (linear scale). The dynamic tube normalization method was selected.

#### Metagenome sequencing (Miseq)

For high‐throughput sequencing, 50 ng of DNA of each of the three WT and GM soil were fragmented by ‘tagmentation’ according to manufacturer's protocol (Nextera DNA sample prep, Illumina) in a final reaction volume of 50 μL. The indexes were then added by PCR according to the Illumina protocol (Nextera Index kit). Libraries (1 μL) were checked on the Bioanalyzer (Agilent, Santa Clara, CA) with High Sensitivity kit. The sequencing was then performed with the Miseq sequencer 2 × 250 bp (v2 chemistry) at École Centrale de Lyon. Sequences were used as singleton, and the 12 data sets were then trimmed (Table S6) with CLC Genomics Workbench 8.5.1 (CLC bio Genomics, Qiagen, Aarhus, Denmark) and mapped against reference sequences (see NGS data analysis section).

#### Phylogenetic Microarrays

The *16S rRNA* genes of soil DNA were amplified using the primers pA and T7‐pH. The forward primer contained a T7 promoter site at the 5′end (5′‐TAATACGACTCACTATAG‐3′), which enabled T7 RNA polymerase mediated *in vitro* transcription using PCR products as templates. Data analysis of microarrays was performed as previously described (Bodrossy *et al*., 2003; Sanguin *et al*., 2006). Probe sequences are available on the website: http://www.genomenviron.org/Research/Microarrays.html, last visited 05/2017).

#### 16S rRNA sequencing (454)

The Beckman recommended primers (16S‐0515F and 16S‐1061R with a MID tag per sample on both primers) targeting the V4‐V6 region were used to obtain 560 bp products from extracted soil DNA and sequenced (Beckman Coulter Genomics). Data were trimmed (Table S6) and analysed according to QIIME pipeline (Caporaso *et al*., 2010). Data were submitted to MG‐RAST website (Bergheim project, http://metagenomics.anl.gov/linkin.cgi?project=mgp12555).

#### Metagenomics analyses

Miseq and Hiseq data (28 metagenomes) were submitted to MG‐RAST website and made publicly available (IMAGMO project, http://metagenomics.anl.gov/linkin.cgi?project=mgp18015). Sample name correspondence is provided in Figure S5. The representative hit classification table with a cut‐off at 80% was downloaded at the genus level and further analysed with STAMP software (Parks *et al*., 2014) version 2.1.3 and MEGAN version 4.70.4 (Huson *et al*., 2011).

### Viral genomic study

#### RNA extraction, cDNA amplification and high‐throughput sequencing

The NGS method of choice was an RNAseq‐based experiment. This method allows the sequencing of any poly‐A‐tailed RNA in the sample. From this, a sanitary status inspection of our testing system was performed. We also checked for potential movement of the transgenic molecule intraplant. As previously mentioned, four categories of samples (GMR, ScGM, WTR and ScWT) were tested. We focused on the GMR transgenic line G68 and ScGM scion grafted onto this line. Total RNA was extracted from 100 mg of leaf tissue using the RNeasy Plant mini kit (Qiagen), as per manufacturer's recommendations, from 6 different WTR, 6 ScWT as well as 5 GMR and 5 ScGM (Figure S5). Inflorescences were also sampled and extracted separately and then samples from the same category were mixed at a 1:1 ratio and that mix was sequenced and renamed WTRi, ScWTi, GMRi and ScGMi. The cDNA libraries were then made at the GeT‐Genotoul platform facility (INRA‐Toulouse, France), using TruSeq Stranded mRNA sample prep kit with in‐house modifications. Experiments were performed on an Illumina Hiseq 3000 (Illumina, San Diego, CA) using a paired‐end read length of 2x150pb with the Illumina Hiseq3000/4000 SBS sequencing kits.

On a second set of samples, a method focusing solely on encapsidated GFLV sequences IC step (Immuno Capture) followed by an RT‐PCR step (Reverse Transcription‐Polymerase Chain reaction) was performed. Polyclonal antibody @GFLV (from our lab) was used at 1:1000 dilution as previously described (Vigne *et al*., 2004b). RT‐PCR was performed using the LongRange (2 step) RT‐PCR kit (Qiagen), amplifying most of the RNA2 molecule. Post‐IC and RNA extraction, some samples from the same category were mixed at a 1:1 ratio (Figure S5) prior to sequencing being performed at 2 × 250 bp on a MiSeq250.

#### NGS data analyses

Analyses of data sets were performed using CLC Genomics Workbench 8.5.1 software (Qiagen). After trimming procedure and quality check, only reads above 70 nucleotides were kept (see Table S6). Commonly, for transgenic reads detection, very stringent parameters (nonetheless allowing for potential PCR and sequencing errors) were used with length fraction of 0.97 and similarity of 0.99. In a second phase, for sanitary status examination [Table S7 (Martelli, 2014)] and variant detection, less stringent parameters were used to detect a maximum diversity of the viral population using length fraction of 0.5 with similarity of 0.7. This was performed after removal of reads corresponding to the transgenic *cp* sequence from each GMR samples.

For the genetic diversity study, ‘*de novo* assembly tool’ from the Workbench 8.5.1 software was used, and the list of contigs obtained was then mapped against RNA1, RNA2 and satRNA complete GFLV consensus genomes, allowing the identification of GFLV contigs.

#### Sequences analyses, genetic diversity and recombination detection

Alignment analysis and tentative maximum likelihood‐based phylogenetic trees were performed using MUSCLE (Edgar, 2004) and MEGA7 (Kumar *et al*., 2016) software. The best ML‐fitted model for each sequence alignment was used, and bootstrapping analyses of 100 replicates were conducted. Genetic diversity (π) of the viral populations was estimated using the Kimura 2‐parameter model, with standard errors of each measure based on 100 replicate bootstraps, as implemented in MEGA6 (Tamura *et al*., 2013). The variation of π along the GFLV genome was evaluated by sliding window analyses using DnaSP v. 5.10 (Librado and Rozas, 2009). The difference between nonsynonymous (*d*_*N*_) and synonymous (*d*_*S*_) substitutions over the coding sequences from GFLV populations was estimated by the Pamilo‐Bianchi‐Li method as implemented in MEGA6. Differences in nucleotide diversity of the viral populations between modalities were tested by analysis of molecular variance (AMOVA), as implemented in Arlequin v. 5.3.1.2 (Excoffier *et al*., 2005). AMOVA calculates the *F*_ST_ index explaining the between‐groups fraction of total genetic diversity. Significance of these differences was obtained by performing 1000 permutations. Tajima's *D* (*D*_T_) and sliding window analyses were conducted using DnaSP v. 5.10 (Librado and Rozas, 2009) in order to distinguish the viral populations evolving randomly (per mutation‐drift equilibrium; *D*_T_  =  0) to those evolving under a nonrandom process (*D*_T_ > 0: balancing selection, sudden population contraction; *D*_T_ < 0: recent selective sweep, population expansion after a recent bottleneck).

All sequences obtained from ‘*de novo* assembly’ analyses were submitted to Recombination Detection Program (RDP v.4.46) (Martin *et al*., 2015) for recombination detection. To confirm the biological occurrence of the recombination, eight sites were tested by RT‐PCR on RNA samples that had been used for RNAseq library construction. Resulting PCR products were Sanger‐sequenced to confirm sequences (Table S5).

#### Statistical analyses

Statistical analyses were performed using the statistical software package Statgraphics Centurion version 15.1.02 (StatPoint technologies Inc., Warrenton, VA) and XLSTAT (v2016‐03‐30882, Addinsoft, Paris, France). When data were not following a normal distribution and/or homoscedastic, both parametric (ANOVA, t‐test) and nonparametric (Kruskal–Wallis, Mann–Whitney) tests were used. While both tests gave similar results and as ANOVA is robust to the partial violation of its assumptions, for simplicity, only ANOVA analyses are presented. *Chi‐squared* statistical tests were performed using website: http://www.socscistatistics.com/tests (last visited 05/2017). Principal component analysis (PCA) and Clustering of PCA (OrdiClust) were computed using Ade4TkGUI package of R software (version 3.2.2) (Thioulouse and Dray, 2007). Statistical significance of groups was evaluated by Monte Carlo test (MC‐Test) with 10 000 iterations.

## Conflict of interest

The authors declare no conflict of interest.

## Supporting information


**Figure S1** Genes targeted for detection by PCR and qPCR experiments.
**Figure S2** Microbial diversity comparison between leaf (RNAseq 2 × 150) and soil samples (Miseq sequencing).
**Figure S3** Phylogenetic relationships of satRNA genomes, GFLV *cp* gene sequences from RNAseq and from IC‐RT‐PCR NGS‐based dataset obtained from GM rootstock (GMR, in blue), non‐GM rootstock (WTR, in red), scion grafted onto GMR (ScGM, in yellow) and scion grafted onto WTR (ScWT, in green) samples assembled with CLC Workbench 8.5.1 software. Phylogenetic tree based on the Maximum likelihood of (a) 31 full‐length satRNA sequences obtained from RNAseq dataset; (b) 75 full‐length GFLV *cp* gene sequences obtained from RNAseq dataset (T is for the transgenic sequence) and (c) 50 full‐length of GFLV *cp* gene sequences obtained from IC‐RT‐PCR dataset. Bootstrap values are shown.
**Figure S4** Genetic diversity analyses of satRNA sequences (a), GFLV *cp* gene sequences (b) and GFLV *cp* gene sequences without clade I sequences (c) all from RNAseq dataset assembled with CLC Workbench 8.5.1 software.
**Figure S5** Map of the greenhouse assay with location of each sample.

**Table S1** Detection of transgenic plant‐derived transcripts from RNAseq (total RNA) and IC‐RT‐PCR‐NGS (virus encapsidated RNA)‐based techniques.
**Table S2** Sanitary status.
**Table S3** SNPs quantification.
**Table S4** *F*_ST_ values for each sampling category.
**Table S5** (part A and B): Crossover sites.
**Table S6** Quality control of DNAseq and RNAseq.
**Table S7** List of reference of grapevine‐infecting viruses and viroids tested for sanitary status investigation with accession number, based on the directory of virus and virus‐like diseases of the grapevine and their agents^62^.

**Appendix S1** Bacterial genomics and soil study. Viral genomic study.
