# Supplementary material for: Metagenomic‐based impact study of transgenic grapevine rootstock on its associated virome and soil bacteriome
Source: Plant Biotechnol J. 2017 Aug 9;16(1):208–20. doi: 10.1111/pbi.12761 (PMC5785345; doi:10.1111/pbi.12761)
Supplement: Supplementary file 1 — Figure S1 Genes targeted for detection by PCR and qPCR experiments. Figure S2 Microbial diversity comparison between leaf (RNAseq 2 × 150) and soil samples (Miseq sequencing). Figure S3 Phylogenetic relationships of satRNA genomes, GFLV cp gene sequences from RNAseq and from IC‐RT‐PCR NGS‐based dataset obtained from GM rootstock (GMR, in blue), non‐GM rootstock (WTR, in red), scion grafted onto GMR (ScGM, in yellow) and scion grafted onto WTR (ScWT, in green) samples assembled with CLC Workbench 8.5.1 software. Phylogenetic tree based on the Maximum likelihood of (a) 31 full‐length satRNA sequences obtained from RNAseq dataset; (b) 75 full‐length GFLV cp gene sequences obtained from RNAseq dataset (T is for the transgenic sequence) and (c) 50 full‐length of GFLV cp gene sequences obtained from IC‐RT‐PCR dataset. Bootstrap values are shown. Figure S4 Genetic diversity analyses of satRNA sequences (a), GFLV cp gene sequences (b) and GFLV cp gene sequences without clade I sequences (c) all from RNAseq dataset assembled with CLC Workbench 8.5.1 software. Figure S5 Map of the greenhouse assay with location of each sample. [file PBI-16-208-s002.pptx]

## Slide 1
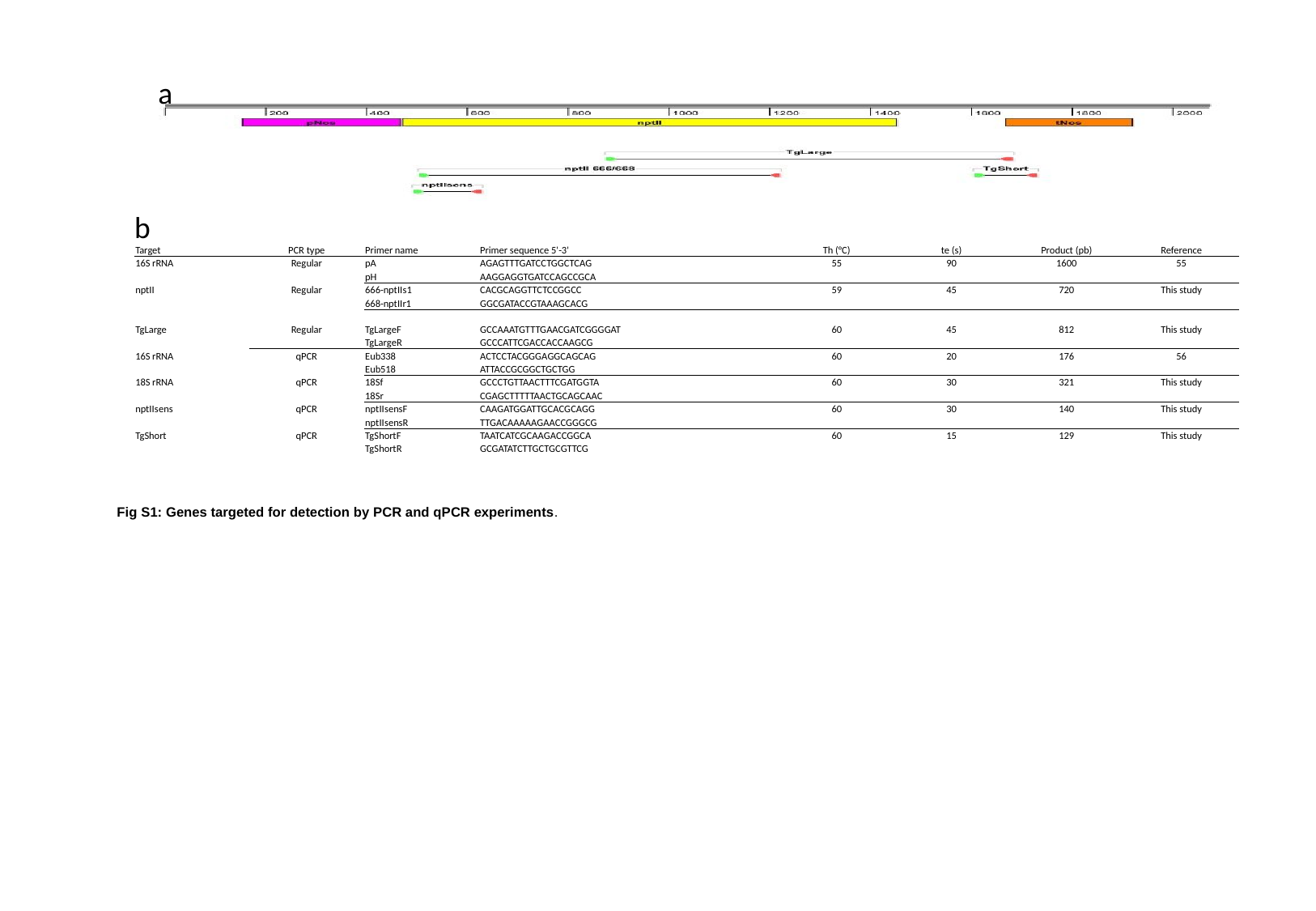

a
b
| Target | PCR type | Primer name | Primer sequence 5'-3' | Th (°C) | te (s) | Product (pb) | Reference |
| --- | --- | --- | --- | --- | --- | --- | --- |
| 16S rRNA | Regular | pA | AGAGTTTGATCCTGGCTCAG | 55 | 90 | 1600 | 55 |
| | | pH | AAGGAGGTGATCCAGCCGCA | | | | |
| nptII | Regular | 666-nptIIs1 | CACGCAGGTTCTCCGGCC | 59 | 45 | 720 | This study |
| | | 668-nptIIr1 | GGCGATACCGTAAAGCACG | | | | |
| TgLarge | Regular | TgLargeF | GCCAAATGTTTGAACGATCGGGGAT | 60 | 45 | 812 | This study |
| | | TgLargeR | GCCCATTCGACCACCAAGCG | | | | |
| 16S rRNA | qPCR | Eub338 | ACTCCTACGGGAGGCAGCAG | 60 | 20 | 176 | 56 |
| | | Eub518 | ATTACCGCGGCTGCTGG | | | | |
| 18S rRNA | qPCR | 18Sf | GCCCTGTTAACTTTCGATGGTA | 60 | 30 | 321 | This study |
| | | 18Sr | CGAGCTTTTTAACTGCAGCAAC | | | | |
| nptIIsens | qPCR | nptIIsensF | CAAGATGGATTGCACGCAGG | 60 | 30 | 140 | This study |
| | | nptIIsensR | TTGACAAAAAGAACCGGGCG | | | | |
| TgShort | qPCR | TgShortF | TAATCATCGCAAGACCGGCA | 60 | 15 | 129 | This study |
| | | TgShortR | GCGATATCTTGCTGCGTTCG | | | | |
 Fig S1: Genes targeted for detection by PCR and qPCR experiments.

## Slide 2
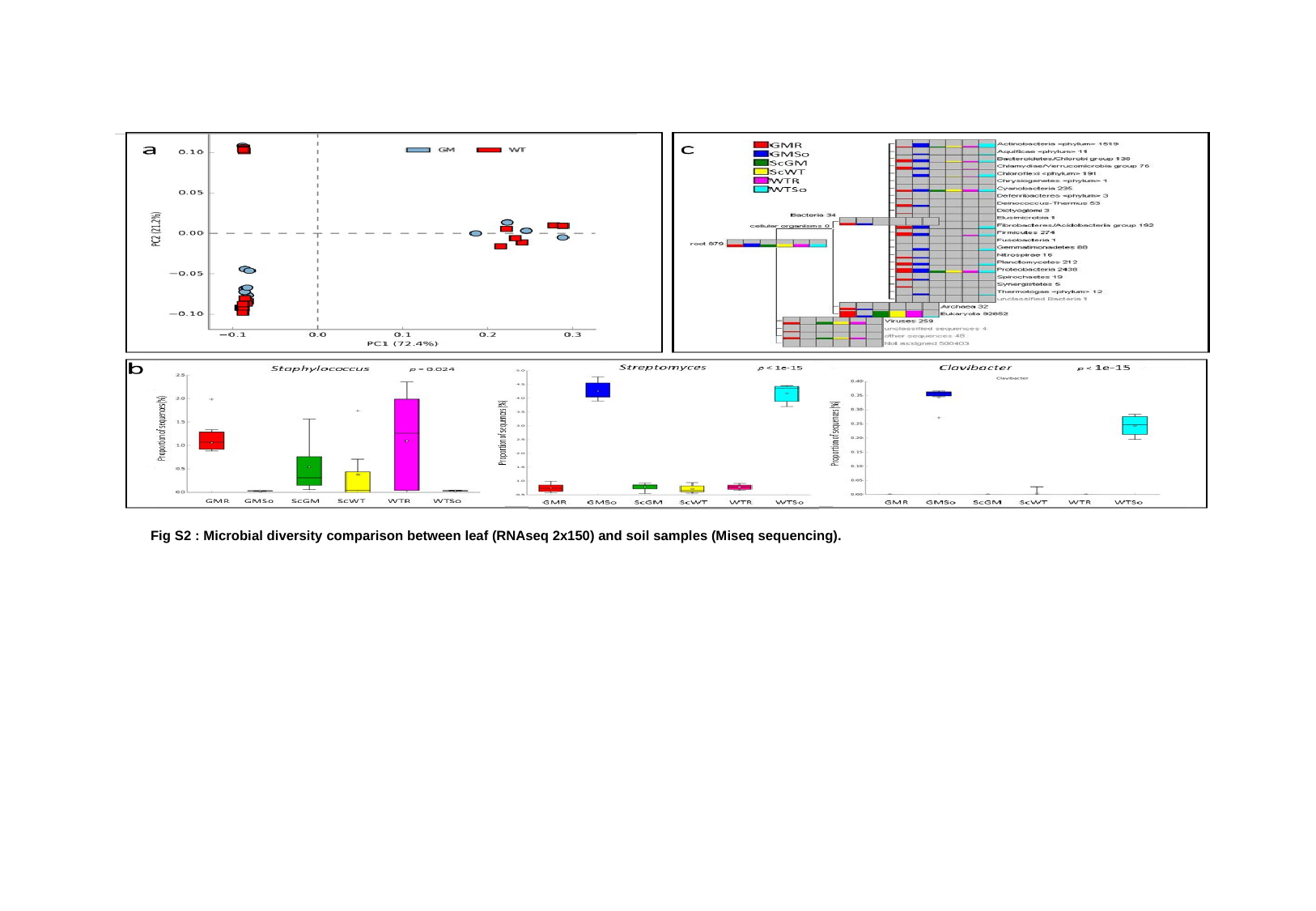

Fig S2 : Microbial diversity comparison between leaf (RNAseq 2x150) and soil samples (Miseq sequencing).

## Slide 3
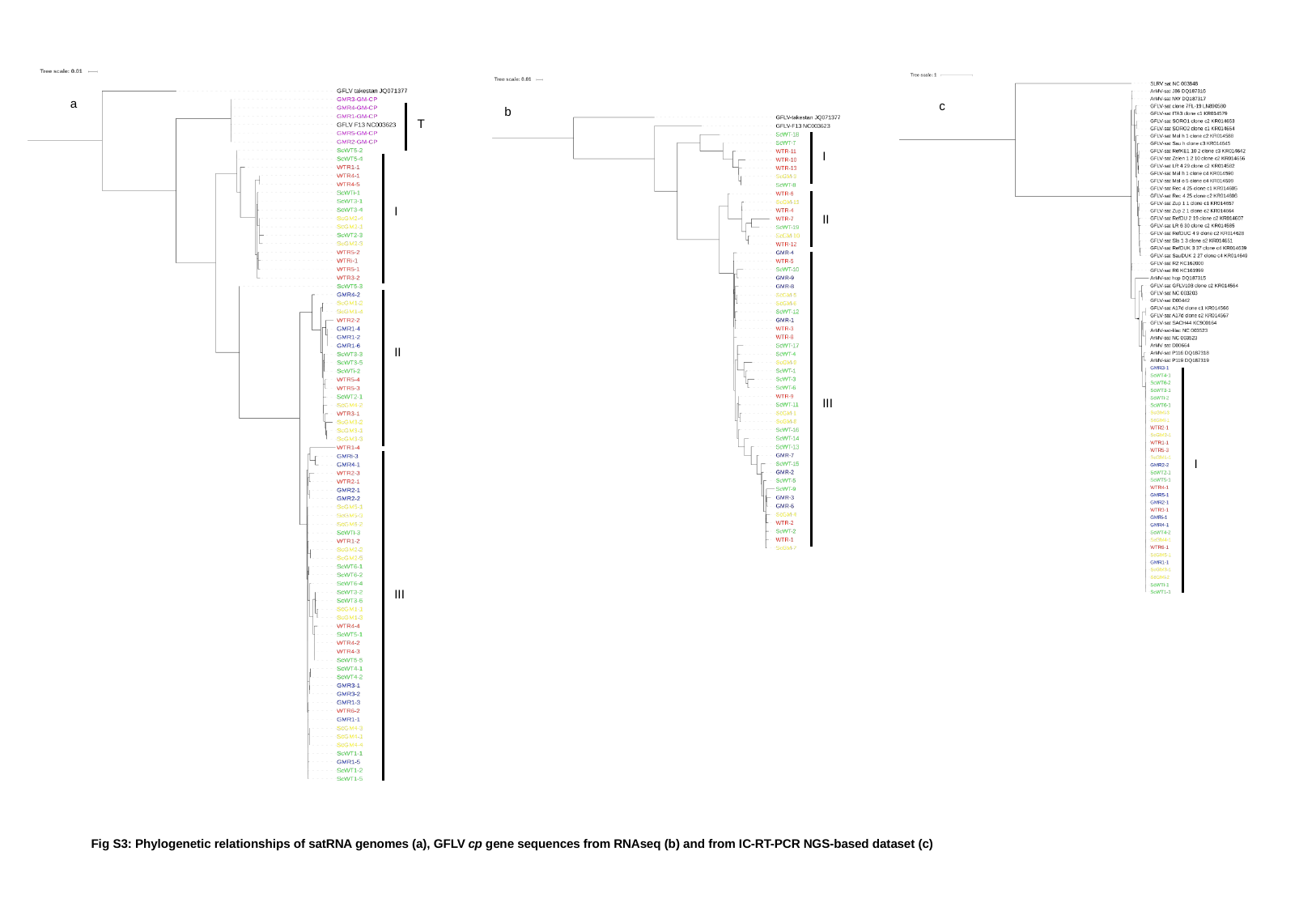

a
T
I
II
III
c
I
b
I
II
III
Fig S3: Phylogenetic relationships of satRNA genomes (a), GFLV cp gene sequences from RNAseq (b) and from IC-RT-PCR NGS-based dataset (c)

## Slide 4
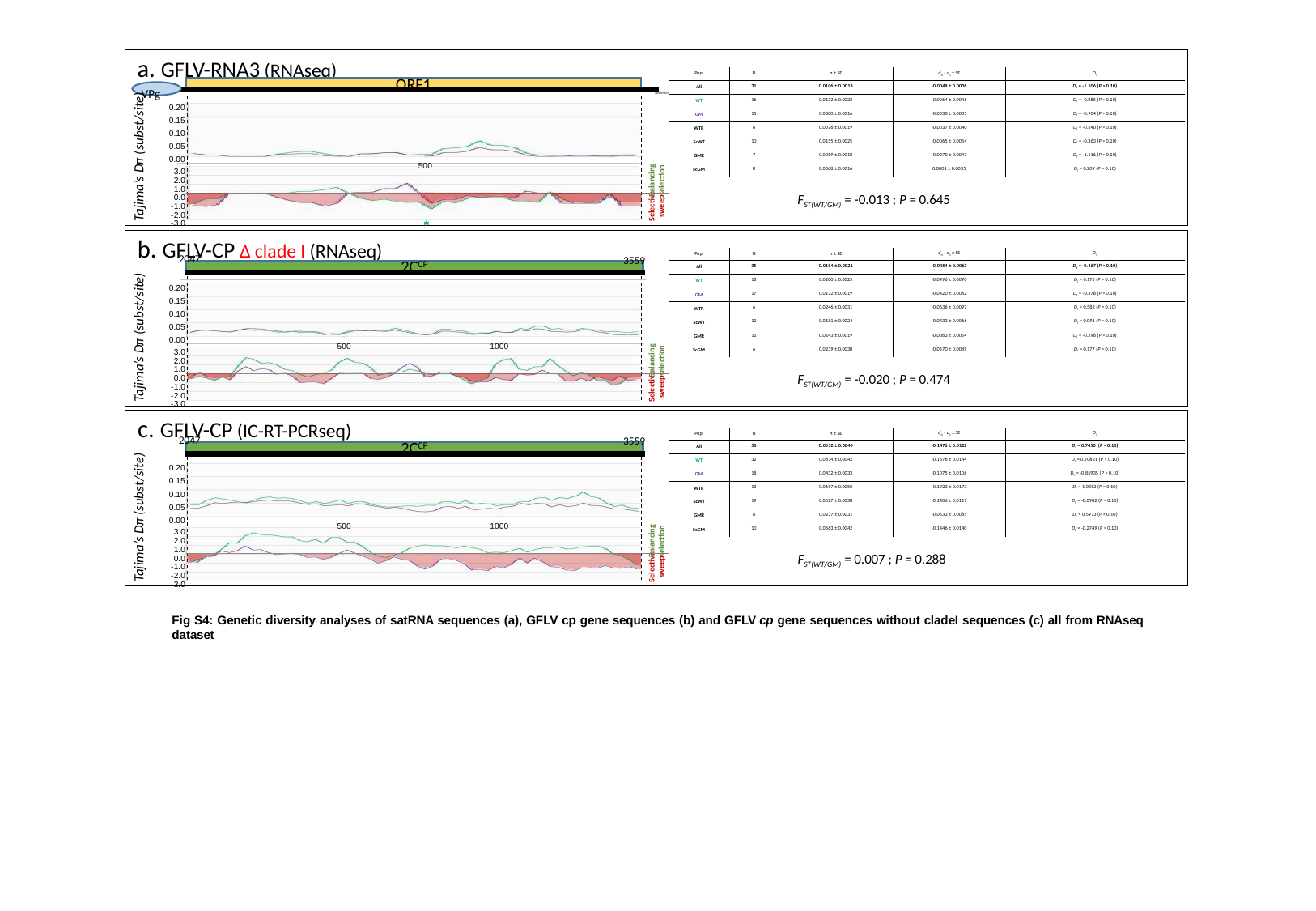

a. GFLV-RNA3 (RNAseq)
| Pop. | N | π ± SE | dN - dS ± SE | DT |
| --- | --- | --- | --- | --- |
| All | 31 | 0.0106 ± 0.0018 | -0.0049 ± 0.0036 | DT = -1.106 (P > 0.10) |
| WT | 16 | 0.0132 ± 0.0022 | -0.0064 ± 0.0046 | DT = -0.885 (P > 0.10) |
| GM | 15 | 0.0080 ± 0.0016 | -0.0030 ± 0.0035 | DT = -0.904 (P > 0.10) |
| WTR | 6 | 0.0076 ± 0.0019 | -0.0037 ± 0.0040 | DT = -0.540 (P > 0.10) |
| ScWT | 10 | 0.0155 ± 0.0025 | -0.0065 ± 0.0054 | DT = -0.363 (P > 0.10) |
| GMR | 7 | 0.0089 ± 0.0018 | -0.0070 ± 0.0041 | DT = -1.116 (P > 0.10) |
| ScGM | 8 | 0.0068 ± 0.0016 | 0.0001 ± 0.0035 | DT = 0.209 (P > 0.10) |
ORF1
VPg
0.20
0.15
π (subst/site)
0.10
0.05
0.00
500
3.0
2.0
Balancing
selection
1.0
Tajima’s D
0.0
-1.0
Selective
sweep
-2.0
-3.0
*
AAAAAA
FST(WT/GM) = -0.013 ; P = 0.645
b. GFLV-CP ∆ clade I (RNAseq)
2047
3559
2CCP
| Pop. | N | π ± SE | dN - dS ± SE | DT |
| --- | --- | --- | --- | --- |
| All | 35 | 0.0184 ± 0.0021 | -0.0454 ± 0.0063 | DT = -0.467 (P > 0.10) |
| WT | 18 | 0.0200 ± 0.0025 | -0.0496 ± 0.0070 | DT = 0.175 (P > 0.10) |
| GM | 17 | 0.0172 ± 0.0019 | -0.0420 ± 0.0062 | DT = -0.378 (P > 0.10) |
| WTR | 6 | 0.0246 ± 0.0031 | -0.0636 ± 0.0097 | DT = 0.582 (P > 0.10) |
| ScWT | 12 | 0.0181 ± 0.0024 | -0.0433 ± 0.0066 | DT = 0.091 (P > 0.10) |
| GMR | 11 | 0.0143 ± 0.0019 | -0.0363 ± 0.0054 | DT = -0.298 (P > 0.10) |
| ScGM | 6 | 0.0239 ± 0.0030 | -0.0570 ± 0.0089 | DT = 0.177 (P > 0.10) |
0.20
0.15
π (subst/site)
0.10
0.05
0.00
500
1000
3.0
2.0
Balancing
selection
1.0
Tajima’s D
0.0
-1.0
Selective
sweep
-2.0
-3.0
FST(WT/GM) = -0.020 ; P = 0.474
c. GFLV-CP (IC-RT-PCRseq)
| Pop. | N | π ± SE | dN - dS ± SE | DT |
| --- | --- | --- | --- | --- |
| All | 50 | 0.0552 ± 0.0040 | -0.1476 ± 0.0122 | DT = 0.7450 (P > 0.10) |
| WT | 32 | 0.0614 ± 0.0042 | -0.1676 ± 0.0144 | DT = 0.70821 (P > 0.10) |
| GM | 18 | 0.0432 ± 0.0033 | -0.1075 ± 0.0106 | DT = -0.85935 (P > 0.10) |
| WTR | 13 | 0.0697 ± 0.0050 | -0.1922 ± 0.0173 | DT = 1.0382 (P > 0.10) |
| ScWT | 19 | 0.0527 ± 0.0038 | -0.1406 ± 0.0117 | DT = -0.0902 (P > 0.10) |
| GMR | 8 | 0.0237 ± 0.0031 | -0.0533 ± 0.0085 | DT = 0.5973 (P > 0.10) |
| ScGM | 10 | 0.0563 ± 0.0042 | -0.1446 ± 0.0140 | DT = -0.2749 (P > 0.10) |
2047
3559
2CCP
0.20
0.15
π (subst/site)
0.10
0.05
0.00
500
1000
3.0
2.0
Balancing
selection
1.0
Tajima’s D
0.0
-1.0
Selective
sweep
-2.0
-3.0
FST(WT/GM) = 0.007 ; P = 0.288
Fig S4: Genetic diversity analyses of satRNA sequences (a), GFLV cp gene sequences (b) and GFLV cp gene sequences without cladeI sequences (c) all from RNAseq dataset

## Slide 5
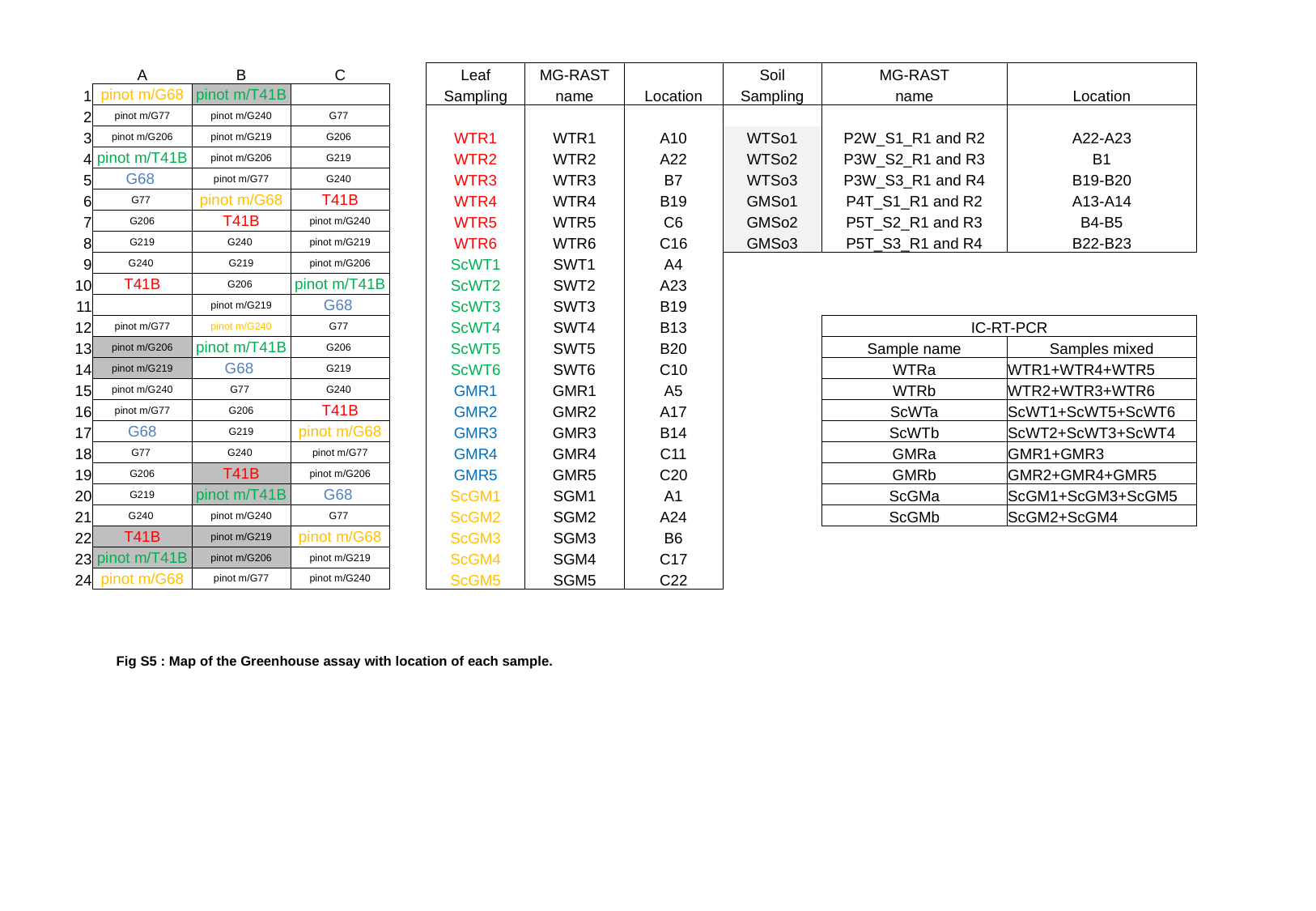

| | A | B | C | | Leaf | MG-RAST | | Soil | MG-RAST | |
| --- | --- | --- | --- | --- | --- | --- | --- | --- | --- | --- |
| 1 | pinot m/G68 | pinot m/T41B | | | Sampling | name | Location | Sampling | name | Location |
| 2 | pinot m/G77 | pinot m/G240 | G77 | | | | | | | |
| 3 | pinot m/G206 | pinot m/G219 | G206 | | WTR1 | WTR1 | A10 | WTSo1 | P2W\_S1\_R1 and R2 | A22-A23 |
| 4 | pinot m/T41B | pinot m/G206 | G219 | | WTR2 | WTR2 | A22 | WTSo2 | P3W\_S2\_R1 and R3 | B1 |
| 5 | G68 | pinot m/G77 | G240 | | WTR3 | WTR3 | B7 | WTSo3 | P3W\_S3\_R1 and R4 | B19-B20 |
| 6 | G77 | pinot m/G68 | T41B | | WTR4 | WTR4 | B19 | GMSo1 | P4T\_S1\_R1 and R2 | A13-A14 |
| 7 | G206 | T41B | pinot m/G240 | | WTR5 | WTR5 | C6 | GMSo2 | P5T\_S2\_R1 and R3 | B4-B5 |
| 8 | G219 | G240 | pinot m/G219 | | WTR6 | WTR6 | C16 | GMSo3 | P5T\_S3\_R1 and R4 | B22-B23 |
| 9 | G240 | G219 | pinot m/G206 | | ScWT1 | SWT1 | A4 | | | |
| 10 | T41B | G206 | pinot m/T41B | | ScWT2 | SWT2 | A23 | | | |
| 11 | | pinot m/G219 | G68 | | ScWT3 | SWT3 | B19 | | | |
| 12 | pinot m/G77 | pinot m/G240 | G77 | | ScWT4 | SWT4 | B13 | | IC-RT-PCR | |
| 13 | pinot m/G206 | pinot m/T41B | G206 | | ScWT5 | SWT5 | B20 | | Sample name | Samples mixed |
| 14 | pinot m/G219 | G68 | G219 | | ScWT6 | SWT6 | C10 | | WTRa | WTR1+WTR4+WTR5 |
| 15 | pinot m/G240 | G77 | G240 | | GMR1 | GMR1 | A5 | | WTRb | WTR2+WTR3+WTR6 |
| 16 | pinot m/G77 | G206 | T41B | | GMR2 | GMR2 | A17 | | ScWTa | ScWT1+ScWT5+ScWT6 |
| 17 | G68 | G219 | pinot m/G68 | | GMR3 | GMR3 | B14 | | ScWTb | ScWT2+ScWT3+ScWT4 |
| 18 | G77 | G240 | pinot m/G77 | | GMR4 | GMR4 | C11 | | GMRa | GMR1+GMR3 |
| 19 | G206 | T41B | pinot m/G206 | | GMR5 | GMR5 | C20 | | GMRb | GMR2+GMR4+GMR5 |
| 20 | G219 | pinot m/T41B | G68 | | ScGM1 | SGM1 | A1 | | ScGMa | ScGM1+ScGM3+ScGM5 |
| 21 | G240 | pinot m/G240 | G77 | | ScGM2 | SGM2 | A24 | | ScGMb | ScGM2+ScGM4 |
| 22 | T41B | pinot m/G219 | pinot m/G68 | | ScGM3 | SGM3 | B6 | | | |
| 23 | pinot m/T41B | pinot m/G206 | pinot m/G219 | | ScGM4 | SGM4 | C17 | | | |
| 24 | pinot m/G68 | pinot m/G77 | pinot m/G240 | | ScGM5 | SGM5 | C22 | | | |
Fig S5 : Map of the Greenhouse assay with location of each sample.
