# Supplementary material for: Metagenomic‐based impact study of transgenic grapevine rootstock on its associated virome and soil bacteriome
Source: Plant Biotechnol J. 2017 Aug 9;16(1):208–20. doi: 10.1111/pbi.12761 (PMC5785345; doi:10.1111/pbi.12761)
Supplement: Supplementary file 2 — Table S1 Detection of transgenic plant‐derived transcripts from RNAseq (total RNA) and IC‐RT‐PCR‐NGS (virus encapsidated RNA)‐based techniques. Table S2 Sanitary status. Table S3 SNPs quantification. Table S4 F ST values for each sampling category. Table S5 (part A and B): Crossover sites. Table S6 Quality control of DNAseq and RNAseq. Table S7 List of reference of grapevine‐infecting viruses and viroids tested for sanitary status investigation with accession number, based on the directory of virus and virus‐like diseases of the grapevine and their agents62. [file PBI-16-208-s001.pptx]

## Slide 1
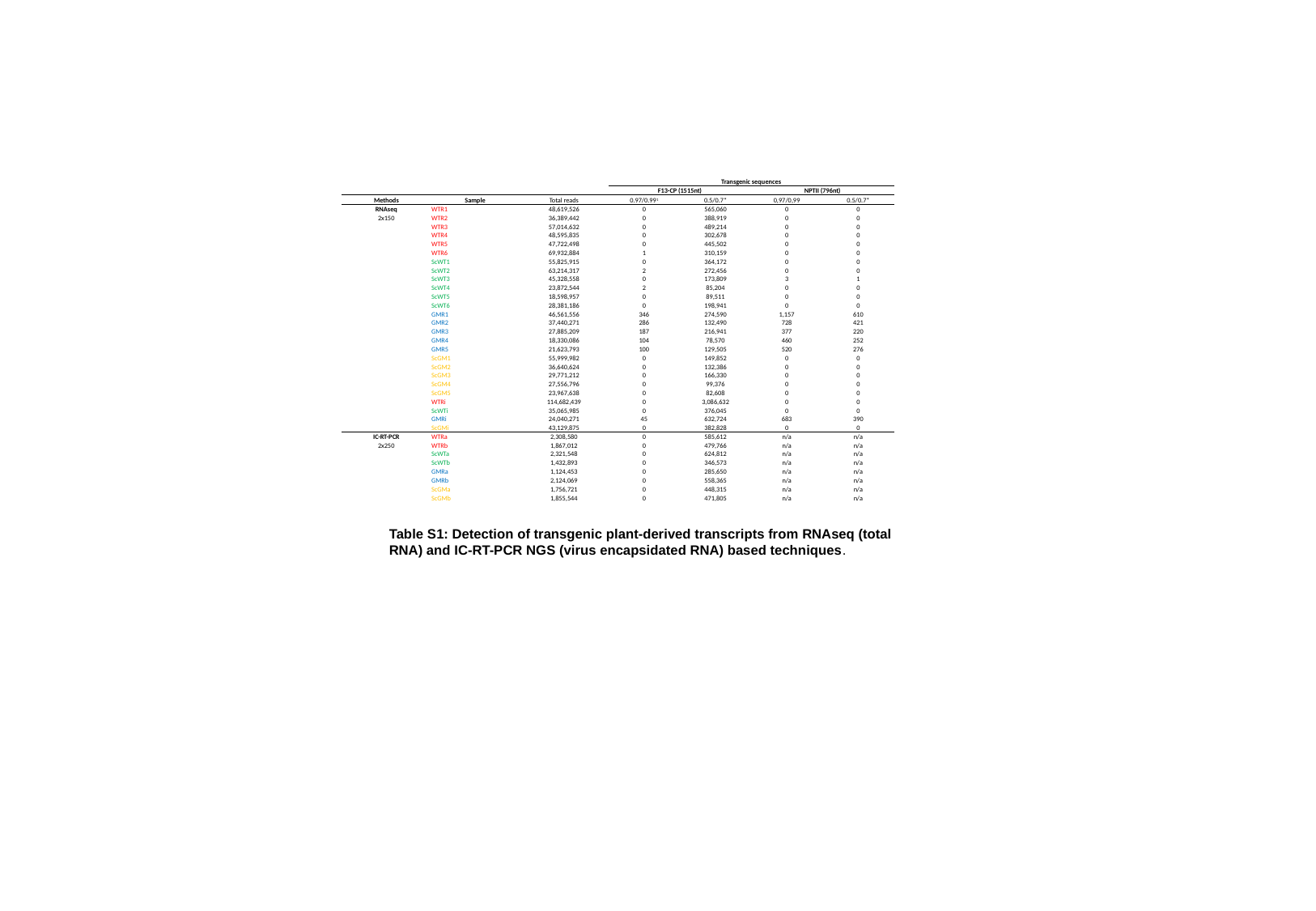

| | | | Transgenic sequences | | | |
| --- | --- | --- | --- | --- | --- | --- |
| | | | F13-CP (1515nt) | | NPTII (796nt) | |
| Methods | Sample | Total reads | 0.97/0.991 | 0.5/0.7\* | 0,97/0,99 | 0.5/0.7\* |
| RNAseq | WTR1 | 48,619,526 | 0 | 565,060 | 0 | 0 |
| 2x150 | WTR2 | 36,389,442 | 0 | 388,919 | 0 | 0 |
| | WTR3 | 57,014,632 | 0 | 489,214 | 0 | 0 |
| | WTR4 | 48,595,835 | 0 | 302,678 | 0 | 0 |
| | WTR5 | 47,722,498 | 0 | 445,502 | 0 | 0 |
| | WTR6 | 69,932,884 | 1 | 310,159 | 0 | 0 |
| | ScWT1 | 55,825,915 | 0 | 364,172 | 0 | 0 |
| | ScWT2 | 63,214,317 | 2 | 272,456 | 0 | 0 |
| | ScWT3 | 45,328,558 | 0 | 173,809 | 3 | 1 |
| | ScWT4 | 23,872,544 | 2 | 85,204 | 0 | 0 |
| | ScWT5 | 18,598,957 | 0 | 89,511 | 0 | 0 |
| | ScWT6 | 28,381,186 | 0 | 198,941 | 0 | 0 |
| | GMR1 | 46,561,556 | 346 | 274,590 | 1,157 | 610 |
| | GMR2 | 37,440,271 | 286 | 132,490 | 728 | 421 |
| | GMR3 | 27,885,209 | 187 | 216,941 | 377 | 220 |
| | GMR4 | 18,330,086 | 104 | 78,570 | 460 | 252 |
| | GMR5 | 21,623,793 | 100 | 129,505 | 520 | 276 |
| | ScGM1 | 55,999,982 | 0 | 149,852 | 0 | 0 |
| | ScGM2 | 36,640,624 | 0 | 132,386 | 0 | 0 |
| | ScGM3 | 29,771,212 | 0 | 166,330 | 0 | 0 |
| | ScGM4 | 27,556,796 | 0 | 99,376 | 0 | 0 |
| | ScGM5 | 23,967,638 | 0 | 82,608 | 0 | 0 |
| | WTRi | 114,682,439 | 0 | 3,086,632 | 0 | 0 |
| | ScWTi | 35,065,985 | 0 | 376,045 | 0 | 0 |
| | GMRi | 24,040,271 | 45 | 632,724 | 683 | 390 |
| | ScGMi | 43,129,875 | 0 | 382,828 | 0 | 0 |
| IC-RT-PCR | WTRa | 2,308,580 | 0 | 585,612 | n/a | n/a |
| 2x250 | WTRb | 1,867,012 | 0 | 479,766 | n/a | n/a |
| | ScWTa | 2,321,548 | 0 | 624,812 | n/a | n/a |
| | ScWTb | 1,432,893 | 0 | 346,573 | n/a | n/a |
| | GMRa | 1,124,453 | 0 | 285,650 | n/a | n/a |
| | GMRb | 2,124,069 | 0 | 558,365 | n/a | n/a |
| | ScGMa | 1,756,721 | 0 | 448,315 | n/a | n/a |
| | ScGMb | 1,855,544 | 0 | 471,805 | n/a | n/a |
Table S1: Detection of transgenic plant-derived transcripts from RNAseq (total RNA) and IC-RT-PCR NGS (virus encapsidated RNA) based techniques.

## Slide 2
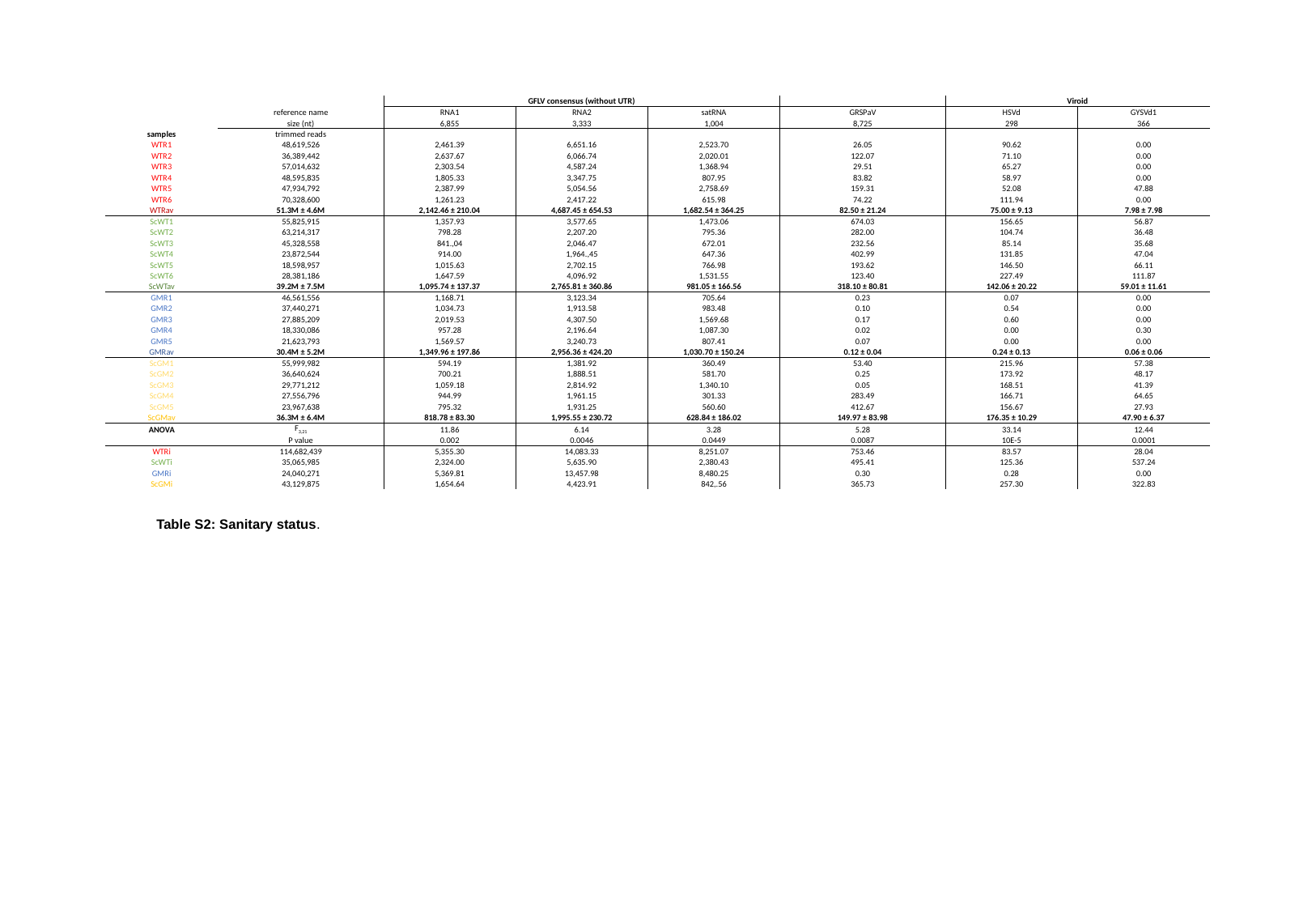

| | | GFLV consensus (without UTR) | | | | Viroid | |
| --- | --- | --- | --- | --- | --- | --- | --- |
| | reference name | RNA1 | RNA2 | satRNA | GRSPaV | HSVd | GYSVd1 |
| | size (nt) | 6,855 | 3,333 | 1,004 | 8,725 | 298 | 366 |
| samples | trimmed reads | | | | | | |
| WTR1 | 48,619,526 | 2,461.39 | 6,651.16 | 2,523.70 | 26.05 | 90.62 | 0.00 |
| WTR2 | 36,389,442 | 2,637.67 | 6,066.74 | 2,020.01 | 122.07 | 71.10 | 0.00 |
| WTR3 | 57,014,632 | 2,303.54 | 4,587.24 | 1,368.94 | 29.51 | 65.27 | 0.00 |
| WTR4 | 48,595,835 | 1,805.33 | 3,347.75 | 807.95 | 83.82 | 58.97 | 0.00 |
| WTR5 | 47,934,792 | 2,387.99 | 5,054.56 | 2,758.69 | 159.31 | 52.08 | 47.88 |
| WTR6 | 70,328,600 | 1,261.23 | 2,417.22 | 615.98 | 74.22 | 111.94 | 0.00 |
| WTRav | 51.3M ± 4.6M | 2,142.46 ± 210.04 | 4,687.45 ± 654.53 | 1,682.54 ± 364.25 | 82.50 ± 21.24 | 75.00 ± 9.13 | 7.98 ± 7.98 |
| ScWT1 | 55,825,915 | 1,357.93 | 3,577.65 | 1,473.06 | 674.03 | 156.65 | 56.87 |
| ScWT2 | 63,214,317 | 798.28 | 2,207.20 | 795.36 | 282.00 | 104.74 | 36.48 |
| ScWT3 | 45,328,558 | 841.,04 | 2,046.47 | 672.01 | 232.56 | 85.14 | 35.68 |
| ScWT4 | 23,872,544 | 914.00 | 1,964.,45 | 647.36 | 402.99 | 131.85 | 47.04 |
| ScWT5 | 18,598,957 | 1,015.63 | 2,702.15 | 766.98 | 193.62 | 146.50 | 66.11 |
| ScWT6 | 28,381,186 | 1,647.59 | 4,096.92 | 1,531.55 | 123.40 | 227.49 | 111.87 |
| ScWTav | 39.2M ± 7.5M | 1,095.74 ± 137.37 | 2,765.81 ± 360.86 | 981.05 ± 166.56 | 318.10 ± 80.81 | 142.06 ± 20.22 | 59.01 ± 11.61 |
| GMR1 | 46,561,556 | 1,168.71 | 3,123.34 | 705.64 | 0.23 | 0.07 | 0.00 |
| GMR2 | 37,440,271 | 1,034.73 | 1,913.58 | 983.48 | 0.10 | 0.54 | 0.00 |
| GMR3 | 27,885,209 | 2,019.53 | 4,307.50 | 1,569.68 | 0.17 | 0.60 | 0.00 |
| GMR4 | 18,330,086 | 957.28 | 2,196.64 | 1,087.30 | 0.02 | 0.00 | 0.30 |
| GMR5 | 21,623,793 | 1,569.57 | 3,240.73 | 807.41 | 0.07 | 0.00 | 0.00 |
| GMRav | 30.4M ± 5.2M | 1,349.96 ± 197.86 | 2,956.36 ± 424.20 | 1,030.70 ± 150.24 | 0.12 ± 0.04 | 0.24 ± 0.13 | 0.06 ± 0.06 |
| ScGM1 | 55,999,982 | 594.19 | 1,381.92 | 360.49 | 53.40 | 215.96 | 57.38 |
| ScGM2 | 36,640,624 | 700.21 | 1,888.51 | 581.70 | 0.25 | 173.92 | 48.17 |
| ScGM3 | 29,771,212 | 1,059.18 | 2,814.92 | 1,340.10 | 0.05 | 168.51 | 41.39 |
| ScGM4 | 27,556,796 | 944.99 | 1,961.15 | 301.33 | 283.49 | 166.71 | 64.65 |
| ScGM5 | 23,967,638 | 795.32 | 1,931.25 | 560.60 | 412.67 | 156.67 | 27.93 |
| ScGMav | 36.3M ± 6.4M | 818.78 ± 83.30 | 1,995.55 ± 230.72 | 628.84 ± 186.02 | 149.97 ± 83.98 | 176.35 ± 10.29 | 47.90 ± 6.37 |
| ANOVA | F3,21 | 11.86 | 6.14 | 3.28 | 5.28 | 33.14 | 12.44 |
| | P value | 0.002 | 0.0046 | 0.0449 | 0.0087 | 10E-5 | 0.0001 |
| WTRi | 114,682,439 | 5,355.30 | 14,083.33 | 8,251.07 | 753.46 | 83.57 | 28.04 |
| ScWTi | 35,065,985 | 2,324.00 | 5,635.90 | 2,380.43 | 495.41 | 125.36 | 537.24 |
| GMRi | 24,040,271 | 5,369.81 | 13,457.98 | 8,480.25 | 0.30 | 0.28 | 0.00 |
| ScGMi | 43,129,875 | 1,654.64 | 4,423.91 | 842,.56 | 365.73 | 257.30 | 322.83 |
Table S2: Sanitary status.

## Slide 3
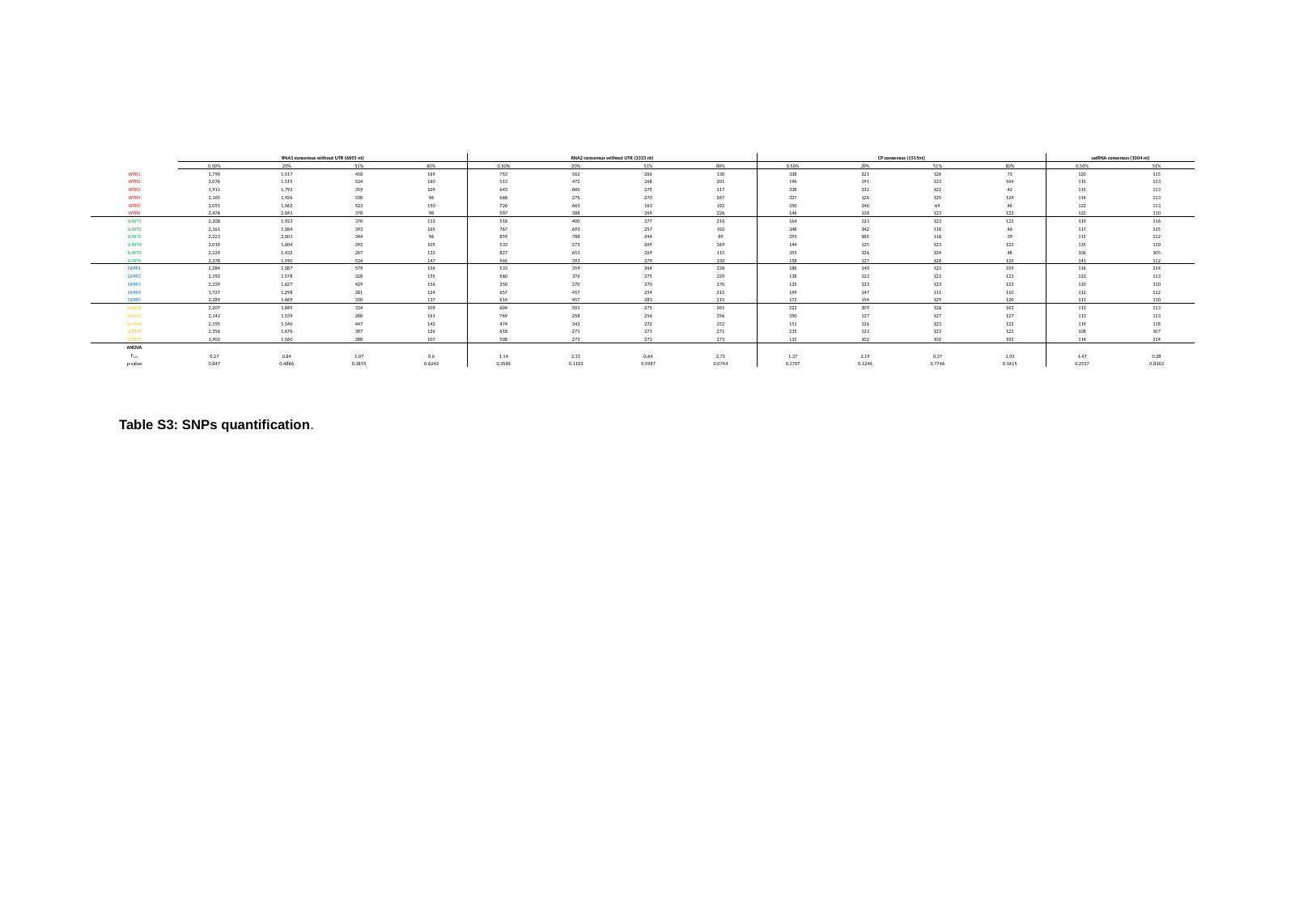

| | RNA1 consensus without UTR (6855 nt) | | | | RNA2 consensus without UTR (3333 nt) | | | | CP consensus (1515nt) | | | | satRNA consensus (1004 nt) | |
| --- | --- | --- | --- | --- | --- | --- | --- | --- | --- | --- | --- | --- | --- | --- |
| | 0.50% | 20% | 51% | 80% | 0.50% | 20% | 51% | 80% | 0.50% | 20% | 51% | 80% | 0.50% | 51% |
| WTR1 | 1,790 | 1,517 | 458 | 169 | 753 | 562 | 266 | 130 | 338 | 221 | 126 | 73 | 120 | 115 |
| WTR2 | 2,076 | 1,515 | 524 | 160 | 513 | 472 | 268 | 201 | 196 | 191 | 123 | 104 | 115 | 113 |
| WTR3 | 1,911 | 1,792 | 359 | 109 | 643 | 605 | 275 | 117 | 338 | 332 | 122 | 43 | 115 | 113 |
| WTR4 | 2,165 | 1,926 | 330 | 96 | 668 | 275 | 270 | 267 | 327 | 126 | 125 | 124 | 114 | 113 |
| WTR5 | 2,055 | 1,562 | 523 | 150 | 726 | 665 | 163 | 102 | 350 | 346 | 69 | 46 | 122 | 113 |
| WTR6 | 2,476 | 2,041 | 378 | 98 | 507 | 388 | 269 | 226 | 146 | 128 | 123 | 122 | 122 | 110 |
| ScWT1 | 2,208 | 1,923 | 370 | 112 | 518 | 400 | 277 | 216 | 164 | 123 | 123 | 123 | 119 | 116 |
| ScWT2 | 2,161 | 1,584 | 393 | 165 | 767 | 693 | 257 | 102 | 348 | 342 | 118 | 46 | 117 | 115 |
| ScWT3 | 2,223 | 2,001 | 344 | 96 | 859 | 788 | 244 | 89 | 393 | 385 | 116 | 39 | 115 | 112 |
| ScWT4 | 2,010 | 1,604 | 292 | 105 | 533 | 273 | 269 | 269 | 144 | 125 | 123 | 123 | 135 | 110 |
| ScWT5 | 2,129 | 1,432 | 267 | 132 | 827 | 653 | 269 | 115 | 355 | 326 | 124 | 48 | 106 | 105 |
| ScWT6 | 2,278 | 1,590 | 524 | 147 | 565 | 393 | 279 | 232 | 158 | 127 | 126 | 125 | 141 | 112 |
| GMR1 | 2,284 | 1,587 | 579 | 156 | 533 | 359 | 264 | 220 | 186 | 140 | 123 | 109 | 116 | 114 |
| GMR2 | 2,192 | 1,578 | 328 | 155 | 560 | 376 | 275 | 229 | 138 | 123 | 123 | 123 | 122 | 113 |
| GMR3 | 2,239 | 1,627 | 429 | 156 | 350 | 270 | 270 | 270 | 125 | 123 | 123 | 123 | 110 | 110 |
| GMR4 | 1,727 | 1,298 | 281 | 124 | 657 | 457 | 254 | 215 | 199 | 147 | 111 | 110 | 112 | 112 |
| GMR5 | 2,289 | 1,669 | 330 | 137 | 614 | 457 | 283 | 215 | 172 | 154 | 129 | 120 | 111 | 110 |
| ScGM1 | 2,207 | 1,845 | 334 | 109 | 604 | 501 | 275 | 201 | 223 | 209 | 126 | 103 | 113 | 113 |
| ScGM2 | 2,142 | 1,539 | 288 | 161 | 769 | 258 | 256 | 256 | 350 | 127 | 127 | 127 | 113 | 113 |
| ScGM3 | 2,195 | 1,540 | 447 | 142 | 474 | 343 | 272 | 252 | 151 | 126 | 123 | 122 | 119 | 118 |
| ScGM4 | 2,356 | 1,676 | 307 | 126 | 618 | 271 | 271 | 271 | 235 | 123 | 123 | 123 | 108 | 107 |
| ScGM5 | 1,903 | 1,560 | 288 | 107 | 508 | 273 | 273 | 273 | 115 | 102 | 102 | 102 | 114 | 114 |
| ANOVA | | | | | | | | | | | | | | |
| F3,21 | 0.27 | 0.84 | 1.07 | 0.6 | 1.14 | 2.31 | 0.64 | 2.73 | 1.37 | 2.19 | 0.37 | 1.93 | 1.47 | 0.28 |
| p-value | 0.847 | 0.4886 | 0.3855 | 0.6242 | 0.3585 | 0.1103 | 0.5987 | 0.0744 | 0.1707 | 0.1246 | 0.7746 | 0.1615 | 0.2557 | 0.8363 |
Table S3: SNPs quantification.

## Slide 4
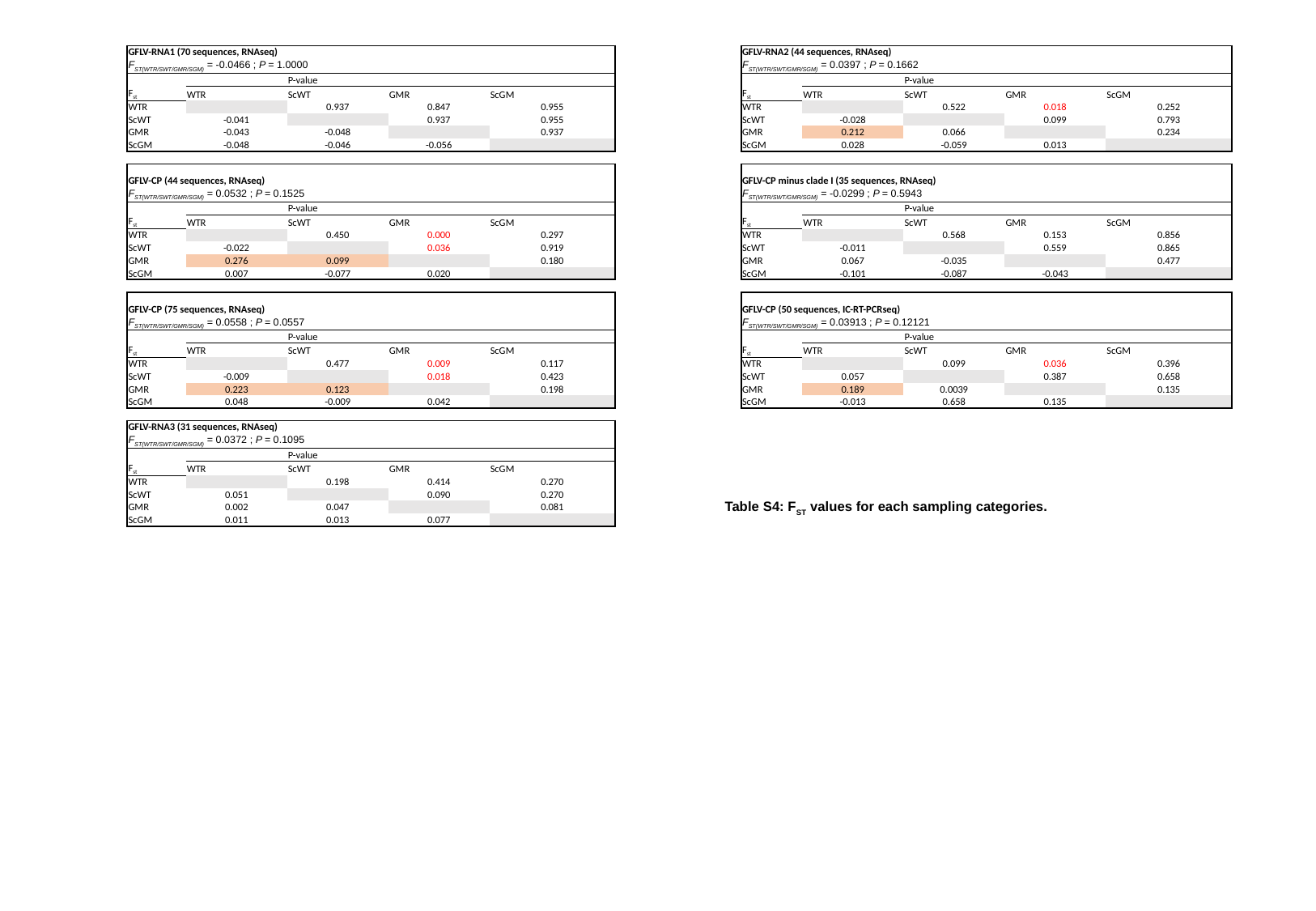

| GFLV-RNA1 (70 sequences, RNAseq) | | | | | | GFLV-RNA2 (44 sequences, RNAseq) | | | | |
| --- | --- | --- | --- | --- | --- | --- | --- | --- | --- | --- |
| FST(WTR/SWT/GMR/SGM) = -0.0466 ; P = 1.0000 | | | | | | FST(WTR/SWT/GMR/SGM) = 0.0397 ; P = 0.1662 | | | | |
| | | P-value | | | | | | P-value | | |
| Fst | WTR | ScWT | GMR | ScGM | | Fst | WTR | ScWT | GMR | ScGM |
| WTR | | 0.937 | 0.847 | 0.955 | | WTR | | 0.522 | 0.018 | 0.252 |
| ScWT | -0.041 | | 0.937 | 0.955 | | ScWT | -0.028 | | 0.099 | 0.793 |
| GMR | -0.043 | -0.048 | | 0.937 | | GMR | 0.212 | 0.066 | | 0.234 |
| ScGM | -0.048 | -0.046 | -0.056 | | | ScGM | 0.028 | -0.059 | 0.013 | |
| | | | | | | | | | | |
| GFLV-CP (44 sequences, RNAseq) | | | | | | GFLV-CP minus clade I (35 sequences, RNAseq) | | | | |
| FST(WTR/SWT/GMR/SGM) = 0.0532 ; P = 0.1525 | | | | | | FST(WTR/SWT/GMR/SGM) = -0.0299 ; P = 0.5943 | | | | |
| | | P-value | | | | | | P-value | | |
| Fst | WTR | ScWT | GMR | ScGM | | Fst | WTR | ScWT | GMR | ScGM |
| WTR | | 0.450 | 0.000 | 0.297 | | WTR | | 0.568 | 0.153 | 0.856 |
| ScWT | -0.022 | | 0.036 | 0.919 | | ScWT | -0.011 | | 0.559 | 0.865 |
| GMR | 0.276 | 0.099 | | 0.180 | | GMR | 0.067 | -0.035 | | 0.477 |
| ScGM | 0.007 | -0.077 | 0.020 | | | ScGM | -0.101 | -0.087 | -0.043 | |
| | | | | | | | | | | |
| GFLV-CP (75 sequences, RNAseq) | | | | | | GFLV-CP (50 sequences, IC-RT-PCRseq) | | | | |
| FST(WTR/SWT/GMR/SGM) = 0.0558 ; P = 0.0557 | | | | | | FST(WTR/SWT/GMR/SGM) = 0.03913 ; P = 0.12121 | | | | |
| | | P-value | | | | | | P-value | | |
| Fst | WTR | ScWT | GMR | ScGM | | Fst | WTR | ScWT | GMR | ScGM |
| WTR | | 0.477 | 0.009 | 0.117 | | WTR | | 0.099 | 0.036 | 0.396 |
| ScWT | -0.009 | | 0.018 | 0.423 | | ScWT | 0.057 | | 0.387 | 0.658 |
| GMR | 0.223 | 0.123 | | 0.198 | | GMR | 0.189 | 0.0039 | | 0.135 |
| ScGM | 0.048 | -0.009 | 0.042 | | | ScGM | -0.013 | 0.658 | 0.135 | |
| | | | | | | | | | | |
| GFLV-RNA3 (31 sequences, RNAseq) | | | | | | | | | | |
| FST(WTR/SWT/GMR/SGM) = 0.0372 ; P = 0.1095 | | | | | | | | | | |
| | | P-value | | | | | | | | |
| Fst | WTR | ScWT | GMR | ScGM | | | | | | |
| WTR | | 0.198 | 0.414 | 0.270 | | | | | | |
| ScWT | 0.051 | | 0.090 | 0.270 | | | | | | |
| GMR | 0.002 | 0.047 | | 0.081 | | | | | | |
| ScGM | 0.011 | 0.013 | 0.077 | | | | | | | |
Table S4: FST values for each sampling categories.

## Slide 5
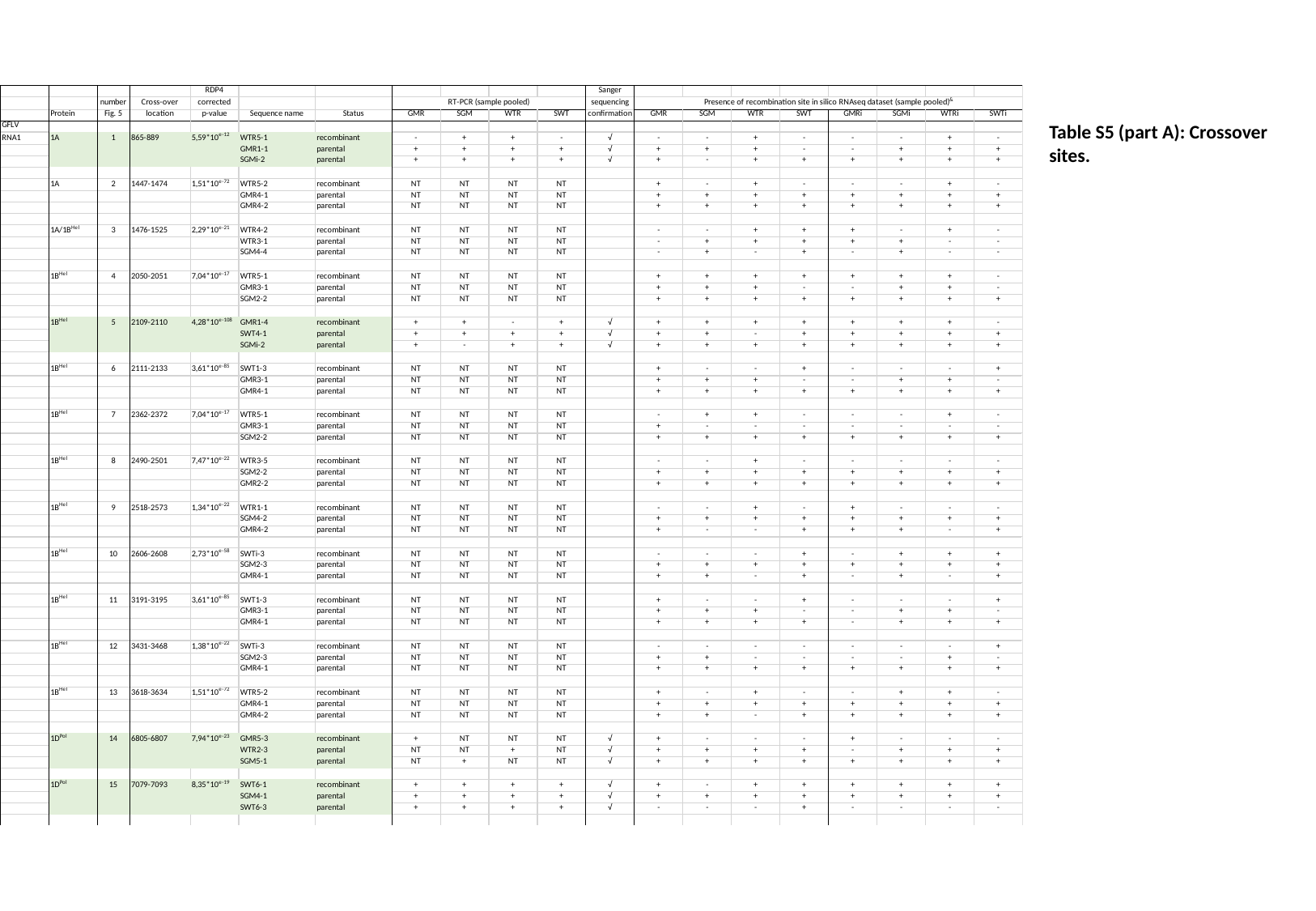

Table S5 (part A): Crossover sites.

## Slide 6
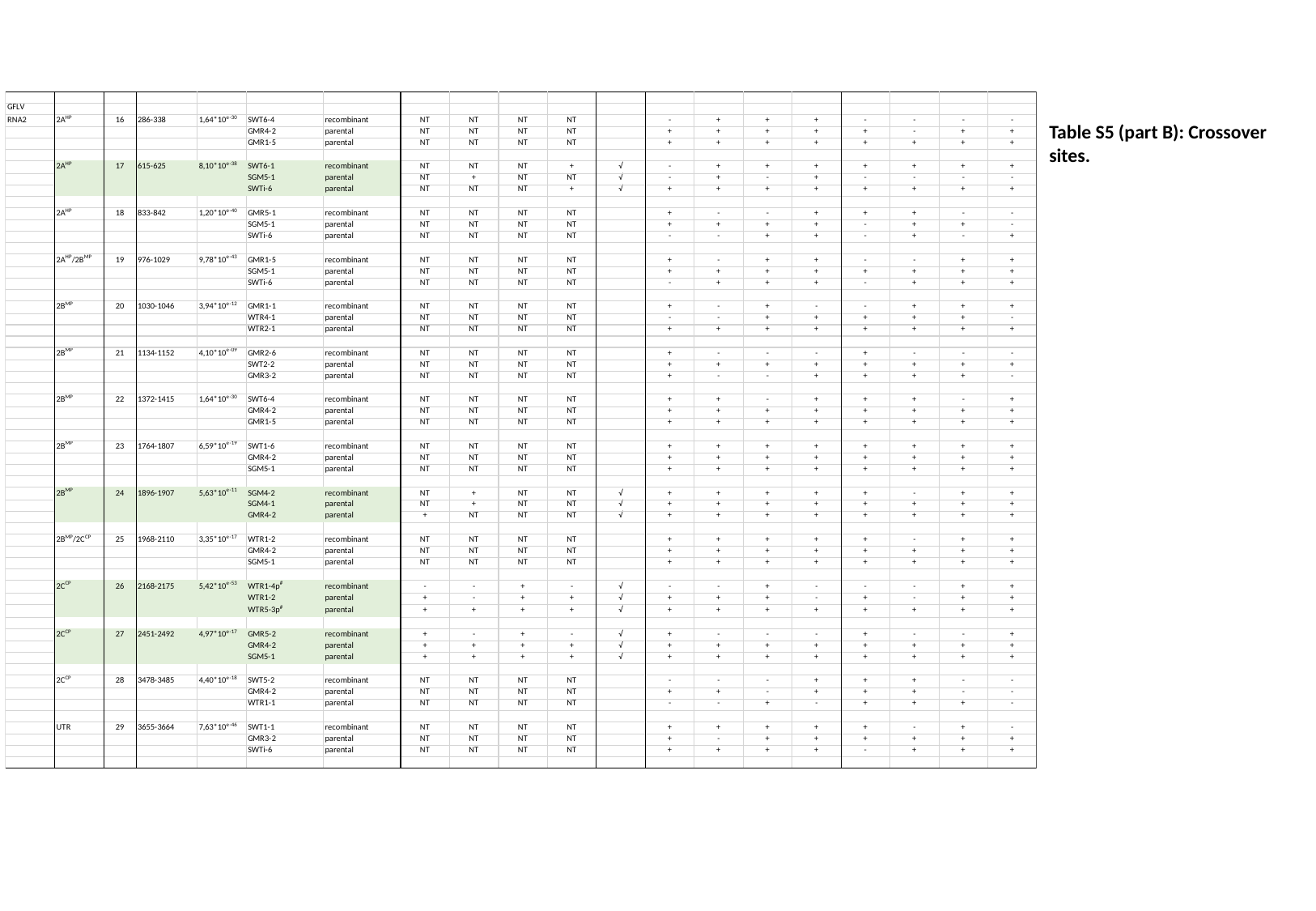

Table S5 (part B): Crossover sites.

## Slide 7
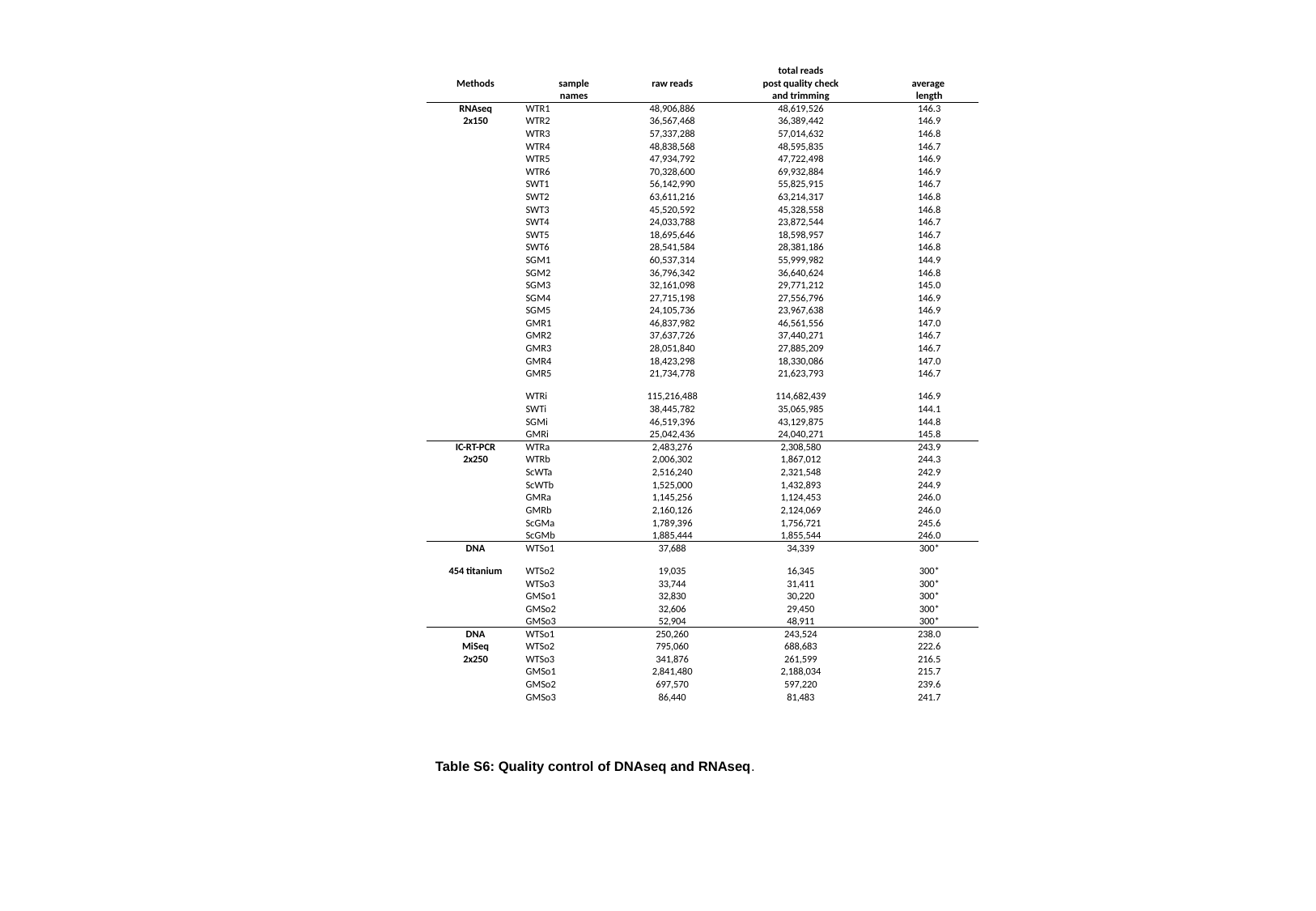

| | | | total reads | |
| --- | --- | --- | --- | --- |
| Methods | sample | raw reads | post quality check | average |
| | names | | and trimming | length |
| RNAseq | WTR1 | 48,906,886 | 48,619,526 | 146.3 |
| 2x150 | WTR2 | 36,567,468 | 36,389,442 | 146.9 |
| | WTR3 | 57,337,288 | 57,014,632 | 146.8 |
| | WTR4 | 48,838,568 | 48,595,835 | 146.7 |
| | WTR5 | 47,934,792 | 47,722,498 | 146.9 |
| | WTR6 | 70,328,600 | 69,932,884 | 146.9 |
| | SWT1 | 56,142,990 | 55,825,915 | 146.7 |
| | SWT2 | 63,611,216 | 63,214,317 | 146.8 |
| | SWT3 | 45,520,592 | 45,328,558 | 146.8 |
| | SWT4 | 24,033,788 | 23,872,544 | 146.7 |
| | SWT5 | 18,695,646 | 18,598,957 | 146.7 |
| | SWT6 | 28,541,584 | 28,381,186 | 146.8 |
| | SGM1 | 60,537,314 | 55,999,982 | 144.9 |
| | SGM2 | 36,796,342 | 36,640,624 | 146.8 |
| | SGM3 | 32,161,098 | 29,771,212 | 145.0 |
| | SGM4 | 27,715,198 | 27,556,796 | 146.9 |
| | SGM5 | 24,105,736 | 23,967,638 | 146.9 |
| | GMR1 | 46,837,982 | 46,561,556 | 147.0 |
| | GMR2 | 37,637,726 | 37,440,271 | 146.7 |
| | GMR3 | 28,051,840 | 27,885,209 | 146.7 |
| | GMR4 | 18,423,298 | 18,330,086 | 147.0 |
| | GMR5 | 21,734,778 | 21,623,793 | 146.7 |
| | WTRi | 115,216,488 | 114,682,439 | 146.9 |
| | SWTi | 38,445,782 | 35,065,985 | 144.1 |
| | SGMi | 46,519,396 | 43,129,875 | 144.8 |
| | GMRi | 25,042,436 | 24,040,271 | 145.8 |
| IC-RT-PCR | WTRa | 2,483,276 | 2,308,580 | 243.9 |
| 2x250 | WTRb | 2,006,302 | 1,867,012 | 244.3 |
| | ScWTa | 2,516,240 | 2,321,548 | 242.9 |
| | ScWTb | 1,525,000 | 1,432,893 | 244.9 |
| | GMRa | 1,145,256 | 1,124,453 | 246.0 |
| | GMRb | 2,160,126 | 2,124,069 | 246.0 |
| | ScGMa | 1,789,396 | 1,756,721 | 245.6 |
| | ScGMb | 1,885,444 | 1,855,544 | 246.0 |
| DNA | WTSo1 | 37,688 | 34,339 | 300\* |
| 454 titanium | WTSo2 | 19,035 | 16,345 | 300\* |
| | WTSo3 | 33,744 | 31,411 | 300\* |
| | GMSo1 | 32,830 | 30,220 | 300\* |
| | GMSo2 | 32,606 | 29,450 | 300\* |
| | GMSo3 | 52,904 | 48,911 | 300\* |
| DNA | WTSo1 | 250,260 | 243,524 | 238.0 |
| MiSeq | WTSo2 | 795,060 | 688,683 | 222.6 |
| 2x250 | WTSo3 | 341,876 | 261,599 | 216.5 |
| | GMSo1 | 2,841,480 | 2,188,034 | 215.7 |
| | GMSo2 | 697,570 | 597,220 | 239.6 |
| | GMSo3 | 86,440 | 81,483 | 241.7 |
Table S6: Quality control of DNAseq and RNAseq.

## Slide 8
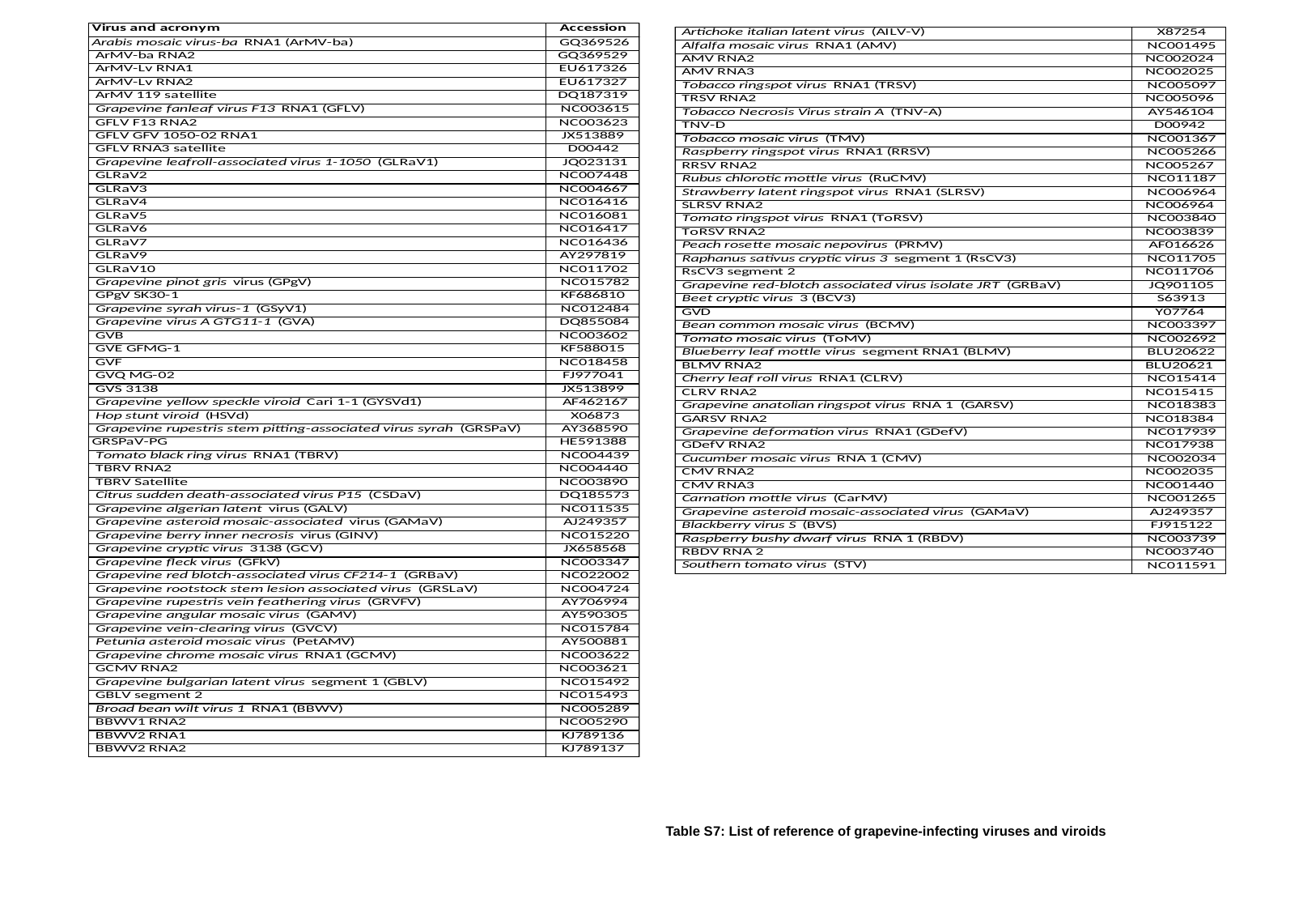

Table S7: List of reference of grapevine-infecting viruses and viroids
