# Supplementary material for: Metagenomic‐based impact study of transgenic grapevine rootstock on its associated virome and soil bacteriome
Source: Plant Biotechnol J. 2017 Aug 9;16(1):208–20. doi: 10.1111/pbi.12761 (PMC5785345; doi:10.1111/pbi.12761)
Supplement: Supplementary file 3 — Appendix S1 Bacterial genomics and soil study. Viral genomic study. [file PBI-16-208-s003.docx]

**Supplemental Experimental procedure.**

**Bacterial genomics and soil study.**

Isolation of antibiotic resistant bacteria

Total cultivable and antibiotic resistant bacteria were isolated from the six soil samples described above. Briefly, one gram of soil was dispersed with 10 mL sodium hexametaphosphate solution (HMP) 0.2% in presence of about 10 glass beads (2-mm diameter) for two hours (CATSSO shaker, maximal speed). Coarse soil particles were eliminated after 30 minutes of sedimentation at 22°C. The supernatant was recovered and centrifuged for 5 min at 8,500 x *g* at 22°C. The pellet was suspended in 1 mL NaCl 0.8%. Soil suspensions were serially diluted and 50µL were spread on ten-fold diluted trypticase soy agar (TSA 1/10) supplemented with 100 µg mL^-1^ cycloheximide and 50 µg mL^-1^ nystatin for total cultivable bacteria counts. For antibiotic resistant bacteria counts, 300 µL of soil suspension were spread on the same medium supplemented with 50 µg mL^-1^ kanamycin and 50 µg mL^-1^ neomycin. Three Petri dishes per dilution were used and soil dispersion was spread in triplicate for each of the 6 soil samples. Petri dishes were incubated for one week at 29°C. Then, 188 (2x94) antibiotic resistant isolates were cultivated on TS broth 1/10 medium supplemented with kanamycin and neomycin (same concentration as mentioned above) for 4 days at 29°C using orbital shaking. Isolates were kept at -80°C in TS broth 1/10 with 25% glycerol.

Bacterial cells recovery

Fifty grams of dry soil were mechanically shaken with 180 mL HMP 0.2% and about 20 glass beads for two hours at 22°C and centrifuged in a swing-bucket rotor for 1 min at 18 x *g* at 10°C. After filtration through sterile gauze, the supernatant was centrifuged for 20 min at 3,220 x *g* at 10°C. The pellet was suspended in 35 mL NaCl 0.8%. Two 50-mL falcon tubes per sample were filled with 11 mL of Nycodenz® solution 1.3 g mL^-1^ and half of the soil suspension was carefully added on the Nycodenz® surface of both tubes. A 40 min centrifugation at 3,220 x *g* at 10°C was performed in a swing-bucket rotor at the lowest setting for acceleration and deceleration in order to separate bacteria from soil particles according to their density. The white bacterial ring (about 4 mL) was recovered at the interface between Nycodenz® and NaCl in both tubes, placed in a single tube filled with NaCl up to 40 mL and centrifuged with a fixed-angle rotor at 9,000 x *g* for 20 min at 10°C. The pellet was then washed with 1 mL NaCl, transferred to a 1.5 mL Eppendorf tube and centrifuged at 13,000 x *g* for 5 min at 10°C. Finally, the pellet was suspended in 50 µL glycerol 10% and kept at -20°C.

PCR amplifications

PCR amplifications were performed using Titanium Taq Polymerase (Clontech Laboratories Inc., CA, USA) on 2 µL of glycerol stock (bacterial isolates or Nycodenz® extraction) or on 20 ng of total DNA in a final volume of 20 µL. PCR products were checked by electrophoresis on a 1% agarose gel. PCR amplifications to prepare standards for qPCR were performed using the Invitrogen Taq Polymerase (Invitrogen, UK) on 20 ng of bacterial strain DNA with appropriate primers (Edwards et al., 1989; Fierer et al., 2005) (Fig S1b). An initial denaturation step at 94°C for 3 min was followed by 30 cycles with denaturation at 94°C for 45s, annealing at temperatures depending on primer set (see Fig S1b), extension at 72°C and a final extension at 72°C for 3 minutes. Size of PCR products were verified by electrophoresis on 2% agarose gels. DNA bands were excised from the gel and purified with the Illustra GFX PCR DNA and gel band purification kits (GE Healthcare, UK). Recovered product concentrations were quantified with Qubit® 2.0 Fluorometer.

Quantitative PCR assays

Quantitative PCR was performed by using the SYBR Green chemistry. The different targeted genes and other information are described in Fig 1 and Table S1. PCR amplifications were performed with SensiMix SYBR No-ROX Kit (Bioline, UK) on DNA (10 ng µL^-1^) and on Nycodenz samples diluted tenfold. Amplifications were carried out using the Rotor-gene 6000^TM^ (QIAGEN, Courtaboeuf, France). The real time PCR (qPCR) reaction mixture yielded a final volume of 20 µL including 1X SensiMix ^TM^ SYBR qPCR Kit, 0.8 µL of each primer at a concentration of 10 µM, 2 µL of DNA (samples and standards), Nycodenz® samples or water (*q.s.p.* 20 µL). qPCR conditions were as follows: initial denaturation step at 95°C for 15 min, then 40 cycles at 95°C for 5s and 60°C for 20s to 45s, then a melt process was run from 50°C to 99°C in order to ensure single product amplification per well. Each sample was run in triplicate.

Metagenome sequencing (Miseq)

For high throughput sequencing, 50ng of DNA of each of the three WT and GM soil were fragmented by ‘tagmentation’ (5 min at 55°C) according to manufacturer’s protocol (Nextera DNA sample prep, Illumina) in a final reaction volume of 50 µL. The tagmented DNAs were then purified with the Zymo-Spin™ kit (Zymo Research, Irvine, USA) up to a final volume of 25 µL. The indexes were then added by PCR (20 µL of tagmented and purified DNA in a final volume of 50 µL) according to the Illumina protocol (Nextera Index kit). PCR products were then purified with the AMPure XP Beads (Beckman Coulter, Brea, USA), eluted in a volume of 32.5 µL of 10 mM Tris pH 8 and concentrations were measured with the Qubit HS kit (Invitrogen) on 2 µL of DNA. Libraries (1 µL) were checked on the Bioanalyzer (Agilent, Santa Clara, USA) with High Sensitivity kit. After normalization at 1 nM, the libraries were pooled (5 µL of each library) and the pool was denatured as per Illumina’s recommendations. The denatured pool was finally diluted to 6 pM in hybridization buffer and combined with a PhiX174 library at a ratio of 1%. The sequencing was then performed with the Miseq sequencer 2x250 bp (v2 chemistry) at École Centrale de Lyon. Sequences were used as singleton and the 12 datasets were then trimmed (Table S6) with CLC Genomics Workbench 8.5.1 (CLC bio Genomics, Qiagen, Aarhus, Denmark) and mapped against reference sequences (see NGS data analyses below).

**Viral genomic study.**

RNA extraction, cDNA amplification and high-throughput sequencing.

The NGS method of choice was an RNAseq-based experiment performed on an Illumina HiSeq3000 using a paired-end read length of 2x150pb. This method allows the sequencing of any poly-A-tailed RNA in the sample. From this, a sanitary status inspection of our testing system was performed, permitting the evaluation of the GFLV population variability in the Bergheim soil. We also checked for potential movement of the transgenic molecule intra-plant. As previously mentioned, four categories of samples (GMR, ScGM, WTR and ScWT) were tested. We focused on the GMR transgenic line G68 and ScGM scion grafted onto this line. Total RNA was extracted from 100 mg of leaf tissue using the RNeasy Plant mini kit (Qiagen), as per manufacturer’s recommendations, from 6 different WTR, 6 ScWT as well as 5 GMR and 5 ScGM (supplementary Fig S5). Inflorescence samples were also sampled and extracted separately and then samples from the same category were mixed at a 1:1 ratio prior to sequencing and renamed WTRi, ScWTi, GMRi and ScGMi. Post extraction, purity criteria (A260/A230 and A260/A280 both >1.8) and quality levels (RIN>8) required for cDNA library preparation for Illumina sequencing were assessed via Nanodrop™ (Thermo Fisher Scientific Inc., Waltham, MA, USA) and Bioanalyzer (Agilent, Santa Clara, CA, USA). The cDNA libraries were then made at the GeT-Genotoul platform facility (INRA-Toulouse, France), using TruSeq Stranded mRNA sample prep kit with in-house modifications. Briefly, the mRNA molecules containing poly(A) were purified using magnetic poly(T) beads from 1.5 μg of each total RNA sample. A fragmentation buffer was added to break the mRNA into short fragments with an average length of 250 bp. From these fragments, the first strands were synthesized using random hexamer primer and the second strands of cDNA were then synthesized. After purification and end repair, these short cDNA were ligated to the sequencing adapters (60 bp on each side) and enriched by polymerase chain reaction (PCR, 10 cycles). Library quality was assessed using the Aati DNF-474 High Sensitivity NGS Kit (Advanced Analytical, Heidelberg, Germany) and then they were quantified by qPCR using the Kapa Library Quantification Kit (Kapa Biosystems Ltd, London, UK). Experiments were performed on an Illumina Hiseq 3000 (Illumina, San Diego, CA, USA) using a paired-end read length of 2x150pb with the Illumina Hiseq3000/4000 SBS sequencing kits.

On a second set of samples, a method focusing solely on encapsidated GFLV sequences IC step (Immunocapture) followed by an RT-PCR step (Reverse Transcription Polymerase Chain reaction) was performed. Polyclonal antibody @GFLV (from our lab) was used at 1:1000 dilution as previously described (Vigne et al., 2004b). RT-PCR was performed using the LongRange (2 step) RT-PCR kit (Qiagen), using primers pair 858F (CCW AAA GTC ATC GCA ATG CTT G) and 860R (GTT ATT TAA ACT TGG TTA TCC CAG) amplifying the 3’ end of the RNA1 molecule and primers pair GFA2-MB-F1 (GCG AAG AGT TTA AGA AAC TCA) and GFA2-MB-R1 (CAC ACA AAA GCG GAA AAC AAG T) amplifying most of the RNA2 molecule. Following IC and RNA extraction, some samples from the same category were mixed at a 1:1 ratio prior to being sequenced at 2x250bp on a MiSeq250. Mixing WTR1 with WTR4 and WTR5 was named WTRa and WTR2 with WTR3 and WTR6 was named WTRb. Mixing ScWT1 with ScWT5 and ScWT6 was named ScWTa, ScWT2 with ScWT3 and ScWT4, is ScWTb. Mixing GMR1 with GMR3 was named GMRa, GMR2 with GMR4 and GMR5, is GMRb. Mixing ScGM1, ScGM3 and ScGM5 was named ScGMa while mixing ScGM2 with ScGM4 was named ScGMb.

NGS data analyses.

Analyses of data sets were performed using CLC Genomics Workbench 8.5.1 software (Qiagen). After trimming procedure and quality check, only reads above 70 nucleotides were kept (see Table S7). For mapping reads to a reference, while all the different costs were always kept the same (cost of mismatch=2, insertion=3, deletion=3, insertion open cost=6, insertion extend cost=1, deletion open cost=6 and deletion extend cost=1), other parameters (e.g. read length and similarity) were adjusted according to each specific inquiry under evaluation. Commonly, for transgenic reads detection, very stringent parameters (nonetheless allowing for potential PCR and sequencing errors) were used with length fraction of 0.97 and similarity of 0.99. In a second phase, for sanitary status examination [Table S7 (Martelli, 2014)] and variant detection, less stringent parameters were used in order to detect a maximum diversity of the viral population using length fraction of 0.5 with similarity of 0.7. This was performed after removal of reads corresponding to the transgenic *cp* sequence from each GMR sample. In order to recover a maximum of sequence diversity, this last analysis was performed using a GFLV consensus sequence obtained from several RNA1, RNA2 and satellite complete GFLV genomes (accession numbers: NC003623, KC900163, JF968121, JQ071374, JQ071377, DQ922679, GQ332368, NC003615, JN391442, GQ332373, JX513894, GQ332372, JF968120, KC900162, DQ187316, DQ187315, DQ187319, DQ187318, DQ187317, NC003523, KC900164, D00442, NC003203, KC162000 and KC161999).
